# Cyclodextrin-assisted photostabilization of 5-fluorouracil: a combined kinetic and computational investigation

**DOI:** 10.1039/d5ra05287d

**Published:** 2025-09-01

**Authors:** Adeela Khurshid, Zubair Anwar, Muneeba Usmani, Reem Altaf, Ayesha Awan, Zuneera Akram, Sadia Hafeez Kazi, Sofia Ahmed, Muhammad Ali Sheraz, Iqbal Ahmad

**Affiliations:** a Department of Pharmaceutics, Baqai Institute of Pharmaceutical Sciences, Baqai Medical University Super Highway, Gadap Road Karachi Pakistan; b Department of Pharmaceutical Chemistry, Baqai Institute of Pharmaceutical Sciences, Baqai Medical University Super Highway, Gadap Road Karachi Pakistan zubair_ana@hotmail.com/zubair_ana@baqai.edu.pk/reem@cust.edu.pk; c Department of Pharmaceutical Chemistry, Faculty of Pharmacy, Capital University of Science and Technology Islamabad Expressway, Kahuta Road, Zone-V Islamabad Pakistan; d Department of Pharmacognosy, Faculty of Pharmaceutical Sciences, Riphah International University G-7/4, 7th Avenue Islamabad Pakistan; e Department of Pharmacology, Baqai Institute of Pharmaceutical Sciences, Baqai Medical University Super Highway, Gadap Road Karachi Pakistan

## Abstract

The photostabilization of 5-fluorouracil (5-FU) has been carried out in the pH range of 2.0–12.0 using cyclodextrins (α-, β-, γ-) as a complexing agent. The inclusion complex formation between 5-FU and CDs has been evaluated using conductometry, FTIR spectroscopy, NMR spectroscopy, differential scanning calorimetry (DSC), and molecular docking simulations. The loss of absorbance at 266 nm for 5-FU in the presence of CDs indicates its photodegradation. The entrapment of 5-FU in α-, β-, and γ-CDs ranges from 22.0–58.0, 30.0–64.0, and 42.0–77.0%, respectively, indicating the formation of inclusion complexes of CDs with 5-FU. The values of Stern–Volmer fluorescence quenching constants and binding constants for α-, β- and γ-CDs are 1.10, 1.69, 3.06 × 10^3^ L mol^−1^ and 2.89, 3.11, 3.62 10^3^ L mol^−1^, respectively. The apparent first-order rate constants (*k*_obs_) for the photodegradation of 5-FU in the presence of α-, β-, and γ-CDs are 1.75–5.12, 1.21–4.89, and 0.85–4.69 × 10^3^, min^−1^, respectively. The photochemical interaction (*k*_2_) of 5-FU with α-, β-, and γ-CDs ranges from 0.25–0.49, 0.63–1.47, and 0.79–2.14 M^−1^ min^−1^ in the pH range 2.0–12.0. The mode of photochemical interaction of 5-FU and CDs and the photostabilization of 5-FU is described based on H–atom abstraction by 5-FU from CD and radical–radical recombination. The computational analysis complemented the experimental findings, showing a significant second-order donor–acceptor interaction, particularly LP → π*, between the hydroxyl groups of γ-CD and the carbonyl groups of 5-FU. The natural population analysis further revealed a shift in electron density from γ-CD to 5-FU, indicating a protective charge-transfer mechanism that contributes to the photostabilization of 5-FU.

## Abstract 2

The photostabilization of 5-fluorouracil (5-FU) has been carried out in the pH range of 2.0–12.0 using cyclodextrins (α-, β-, γ-) as a complexing agent.

## Graphical abstract

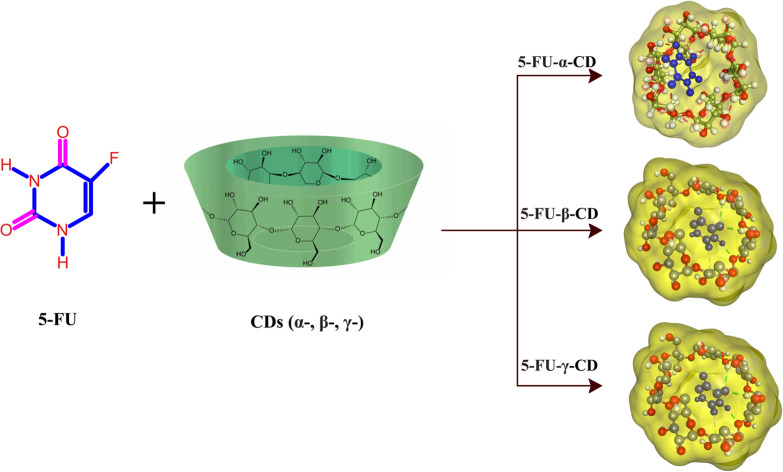

## 1. Introduction

**Fig. 1:**
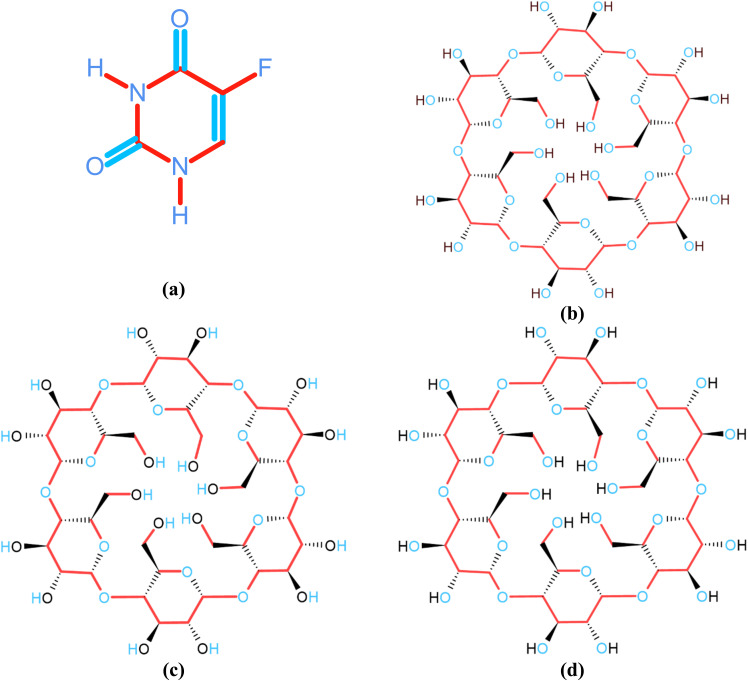
Chemical structures of 5-fluorouracil (5-FU) (a) and cyclodextrins: (b) α-CD, (c) β-CD, (d) γ-CD.

5-Fluorouracil (5-fluoropyrimidine-2,4 (1*H*,3*H*)-dione) (5-FU) (Fig. 1a) is a pyrimidine uracil analogue. It is an antineoplastic agent acting as an antimetabolite and is commonly used for palliative treatment of gastrointestinal cancer.^1–4^ 5-FU undergoes hydrolysis in alkaline solution to form barbituric acid, which is rapidly degraded to other products.^5–9^ It is unstable in aqueous and 5% glucose solutions at room temperature.^10,11^ 5-FU is not ionized in acidic medium and is stable below pH 7 for clinical use.^12,13^ The oxidative degradation of 5-FU in aqueous solution by potassium permanganate has been studied.^14^

5-FU is a photosensitive compound and should be protected from light during storage.^1^ The photodegradation of 5-FU in aqueous and methanolic solutions, as well as in systemic and topical formulations, has been studied and some photoproducts have been identified by thin-layer chromatography, ^1^H and ^13^C NMR, and ESI-mass spectrometry.^15^ The photooxidation processes involved in the degradation of 5-FU by H_2_O_2_, Fe^2+^ and TiO_2_ in aqueous solution and the biodegradability of the identified photoproducts have been reported.^16–19^ The photodegradation of 5-FU by different UV lamps using LC–IT–MS/MS has also been studied and the products identified.^20^ The mechanism of degradation of 5-FU using a polymeric catalyst, graphitic carbon nitride (g-C_3_N_4_) has been investigated.^21^

The nature of products formed on the photodegradation of 5-FU depends on the radiation wavelength/intensity and environmental conditions. Most of these studies are based on the identification of the photoproducts of 5-FU by spectrometric techniques and elucidation of the degradation pathways. The kinetics of its photodegradation over a wide range of pH have not been reported, and the photostabilization of 5-FU using cyclodextrin complexation has not so far been studied.

Cyclodextrins (CDs) (Fig. 1b–d) have been extensively used in the chemical and photostabilization of drug substances, as well as to improve their solubility, formulation, bioavailability, dissolution profile, and delivery.^22–33^ The thermal behavior^34^ and photochemical interactions of CDs inclusion complexes with various compounds such as riboflavin-β-CD,^35^ 1,4-naphthoquinone-α-/β-/γ-CD,^36^ anthraquinone-2,6-disulfonic acid disodium-β-CD,^37^ 1,8-naphthalimide-linker-phenothiazine dyads-β-/γ-CD,^38^ carbonyl compounds-β-CD,^39^ (hydrogen abstraction), acrylamide/benzophenone-Me-β-CD,^40^ toluene-β-CD,^41^ 4-chlorophenol-β-CD,^42^ α-alkyl dibenzyl ketones-β-/γ-CD,^43^ ketoprofen-β-CD,^44^ tolmetin-β-CD^45^ have been studied. CD inclusion complexes of 5-FU have been prepared, characterized, and used to improve its aqueous solubility, dissolution characteristics, and cytotoxic activity.^46–50^ However, no kinetic studies on the photostability of these complexes have been carried out. The object of this work is to conduct a detailed study of the photolysis of 5-FU in a wide range of pH, characterization of 5-FU-CD inclusion complex through FTIR, differential scanning calorimetry (DSC), nuclear magnetic resonance (NMR) spectroscopy, conductometry, computational analysis, and to evaluate the kinetics of its degradation reactions and to make an attempt for its photostabilization by complexation with CDs and propose a mechanism for the reaction.

## 2. Materials and methods

### 2.1 Materials

5-Fluorouracil (99%) (5-FU) was obtained from Sigma Aldrich. All reagents and solvents of the highest purity were procured from Merck. The following buffer systems were used: potassium chloride–hydrochloric acid, pH 2.0; citric acid–disodium phosphate, pH 2.5–8.0; sodium tetraborate–hydrochloric acid, pH 8.5–9.0; sodium tetraborate–sodium hydroxide, pH 9.5–10.5; disodium phosphate–sodium hydroxide, pH 11.0–12.0. The ionic strength of buffer solutions was 0.001 M in each case. Deionized water obtained from the Millipore Milli-Q plus system (Bedford, USA) was used throughout.

### 2.2 pH measurements

The CP-501 Elmetron pH meter, Poland (accuracy ± 0.01 pH units), with a combination electrode was used for the measurement of the pH of solutions. The electrode calibration was carried out using standard buffer solutions of pH 4.0, 7.0, and 9.0.

### 2.3 UV absorption spectrometry

The UV absorption spectra of 5-FU solutions were measured by a Thermo Scientific UV-visible spectrophotometer (Evolution 201) using 1 cm quartz cells.

### 2.4 Spectrofluorometry

The fluorescence emission of 5-FU solutions was measured at 335 nm on excitation at 266 nm^51^ by FP-8300 Jasco Spectrofluorometer at room temperature (25 ± 1 °C). The fluorescence intensity was measured in relative units. A pure 5-FU solution 5 μmol l^−1^ was used as standard.

### 2.5 Light intensity measurements

Potassium ferrioxalate actinometery^52^ was used to measure the intensity of a 30 W Philips TUV Tube, and a value of 5.50 ± 0.50× 10^18^ quanta s^−1^ (9.10 ± 0.95 einstein s^−1^) was obtained.

### 2.6 Conductivity measurements

The molar conductivity of 5-FU solutions (2.0 × 10^−4^ M) was measured in the presence of CDs (0–3.2 mM) by an AD 3000 conductivity meter (ADWA, Hungary) with an attached A76309 conductivity probe. The calibration of the meter was carried out using a standard solution of conductivity 1413 μS cm^−1^ (25 ± 1 °C). The probe was immersed in the calibration solution and the meter was calibrated.

### 2.7 Fourier transform infrared (FTIR) spectroscopy

The Fourier transform infrared (FTIR) spectra were measured by a Nicolet iS5 FTIR spectrometer (Thermo Scientific, USA). The sample was placed on the diamond crystal optical base (iD7 ATR, Thermo Scientific, UK), and the IR spectra were obtained in the wavenumber range of 4000–700 cm^−1^ at a resolution of 4 cm^−1^ after performing 128 scans. The spectra were analyzed using Omnic software (version 9.0).

### 2.8 Preparation of inclusion complexes

5-FU and CDs (α-CD, β-CD, and γ-CD) were weighed in a 1 : 1 molar ratio and dissolved in double-deionized water. These solutions were then freeze-dried in a freeze dryer (9FD-10 MR, Lab Kits, Hong Kong) at −55 °C for 5 h. These samples have been dried at 10 Pa pressure for 16 h.

### 2.9 Differential scanning calorimetry

Differential Scanning Calorimetry (DSC) of the pure (5-FU, α-CD, β-CD, γ-CD) and freeze-dried (5-FU-α-CD, 5-FU-β-CD, 5-FU-γ-CD) samples was carried out using a differential scanning calorimeter (Model DSC 100, Lab-Kits, Hong Kong). Indium and Zinc were used for the calibration of the instrument. Samples (5.0 ± 0.1 mg), accurately weighed in aluminum pans, were heated at a rate of 10 °C per min under a nitrogen atmosphere at a flow rate of 20 ml per min in the range of 30 to 200 °C. The DSC thermograms of each sample were obtained in duplicate. The collected data were analyzed by the built-in software.

### 2.10 Nuclear magnetic resonance (NMR) spectroscopy

^1^H NMR spectra were carried out using Bruker-AV-500 spectrometer (Switzerland) operating at 500 MHz. The solutions of 5-FU (5.0 × 10^−5^ M) and CDs (1.25 × 10^−3^ M) were prepared in D_2_O with a percentage purity of 99.9%.

### 2.11 Computational analysis

#### 2.11.1 Molecular geopmetry optimization

The optimization of target molecules, the initial geometry of 5-FU complexed with γ-CD was constructed using Avogadro 1.2, an open source molecular editor and visualization tool. Gaussian 09W software was used to perform geometric optimization using semi-empirical AM1 method. This was employed to rapidly obtain a reasonable starting geometry while minimizing computational costs.

#### 2.11.2 Mullekin charge analysis (MAC)

The Mulliken atomic charges were calculated directly from the optimized structures using the Hartree–Fock (HF) method with the 6-31G(d) basis set after geometry optimization. The Pop = Full keyword in the Gaussian input was used to perform a Mulliken population analysis, which calculates the charge distribution across the atoms based on the molecular wavefunction. The Mulliken atomic charges were extracted from the Gaussian output and analyzed to understand the electron distribution within the molecule.

#### 2.11.3 Natural bond orbital analysis

A natural population analysis was performed on the optimized geometry using the Hartree–Fock (HF) method with the 6-31G(d) basis set. This approach is widely used for obtaining natural bond orbital data necessary for electron distribution mapping. A single-point energy calculation was carried out with the Pop = NBO keyword to carry out the natural population analysis. The approach partitions the electron density into natural atomic orbitals and provides atomic charges based on electron population distribution. The electron delocalization, polarization, and interaction analysis within the complex were assessed using the resulting natural charges. The second-order perturbation theory was also studied to understand the donor–acceptor interactions. The stabilization energies *E*^2^ obtained from second-order perturbation theory describe the electron delocalization between filled (donor) and empty (acceptor) orbitals. The analysis describes the types of interactions, such as hydrogen bonding, non-covalent interactions, or charge transfer within the molecular complex.

#### 2.11.4 Molecular docking study

A detailed molecular docking study was undertaken to gain a deeper understanding of the complex interactions between 5-FU and CDs (α-, β-, γ-). The interaction between CDs and a ligand was investigated by analyzing their binding energies (kcal mol^−1^) and interaction patterns. Initially, the three-dimensional structure of 5-FU was obtained using the PubChem database. Following that, the process of protonation, depreciation, and charge assignment was carried out on 5-FU. The three-dimensional crystal structure of the α-, β-, and γ-CDs, was obtained from the protein data bank library. The Protein DataBase codes 3WMS, 3CGT, and 4JCM were obtained from the RCSB-PDB database. The α-, β-, and γ-CDs were resolved with a resolution of 2.3 Å, 2.4 Å, and 1.67 Å, respectively. The structures of all three CDs consist of a homodimer of the A chain. Before its docking, the BIOVIA Discovery Studio Visualizer removed previously attached ligand, water, and heterocyclic chains. Polar hydrogen and Kollman charges were attached to the receptor file to generate the PDBQT file using UCSF ChimeraX version 1.7.1 software. The 5-FU was docked with α-, β-, and γ-CDs to complete the interactions using Pyrx virtual screening software. The results of the docking process have been organized carefully and saved in PDB formats.

### 2.12 Photolysis of 5-FU

A 5.0 × 10^−5^ M solution of 5-FU (pH 2.0–12.0) containing 0.25–1.25 × 10^−3^ M α-, β- or γ-CDs was prepared in a 100 ml beaker (Pyrex). It was placed in a thermostat bath (25 ± 1 °C) and irradiated with a 30 W Philips TUV tube (100% emission at 254 nm), fixed horizontally at a distance of 25 cm. Samples of the photolyzed solutions at appropriate intervals were used for the chromatographic assay and the spectral variation determination.

### 2.13 HPLC assay

#### 2.13.1 Chromatographic conditions

The HPLC system (Model LC 10ATVP, Shimadzu, Japan) equipped with a UV detector (Model SPD-10AVP) was connected to a microcomputer. A Purospher RP-8 endcapped column (5 μm) with a mobile phase consisting of a mixture of water and acetonitrile (70 : 30 v/v) at pH 6.0, adjusted with phosphoric acid. The assay was performed at room temperature (25 ± 1 °C) using isocratic conditions. The injection volume was 20 μL, and the flow rate was 1 ml min^−1^. All solutions and the mobile phase were sonicated for 25–30 min before use. The detection of 5-FU was carried out at 266 nm. The assay method has been validated under the experimental conditions used for its application to the photolysis studies of 5-FU.

#### 2.13.2 Method validation

HPLC method for the analysis of 5-FU was validated according to the guidelines of ICH.^53^ The validation parameters include system suitability, linearity, accuracy, precision, the limit of detection (LOD), the limit of quantification (LOQ), and robustness. The details of these parameters are given in the Supplementary Data.

### 2.14 Determination of quantum yield

The quantum yields (*Φ*) of 5-FU on photolysis in the presence of different CDs have been determined by utilizing the value of the intensity (*Q*) of Philips TUV (5.50 ± 0.50 ×10^18^ quanta s^−1^; 9.10 ± 0.95 Einstein s^−1^) and the ratio (*R*) of the area under the emission bands of the TUV absorbed by 5-FU (254 and 313 nm) to the total area of emission bands of the TUV tube.1.0
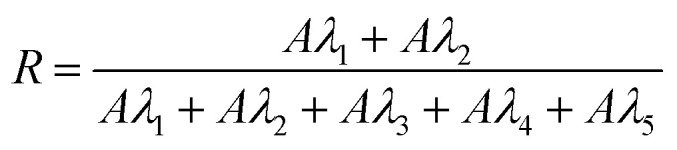
where.

*λ*_1_, *λ*_2_, *λ*_3_, *λ*_4_ and *λ*_5_ are 254, 313, 366, 405 and 436, respectively.2.0
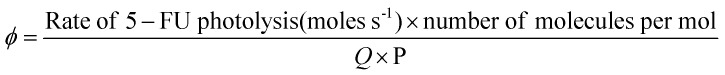


## 3. Results and discussion

### 3.1 Confirmation of purity

Before the analytical method validation, it is important to check the purity of the drug (5-FU) to assess the presence of impurities and degradation products. The purity was confirmed using FTIR spectrometry and the spectrum obtained was compared with the reported^46–49,54^ (Fig. S1). It has been found that there is no difference in the spectra of the reported and the sample, indicating no impurities and degradation products present in the sample (5-FU).

**Fig. 2:**
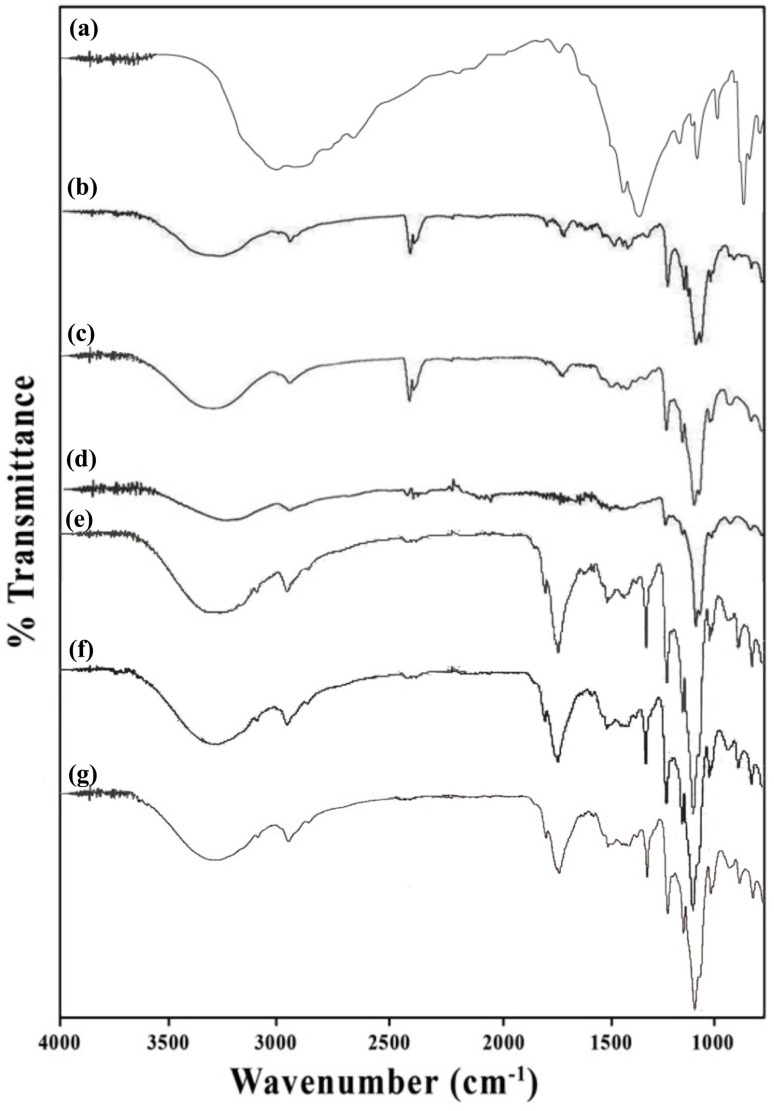
FTIR spectra of 5-FU (a), α-CD (b), β-CD (c), γ-CD (d), 5-FU–α-CD (e), 5-FU-β-CD (f), 5-FU-γ-CD (g).

FTIR spectroscopy has been used to evaluate the interaction between a guest molecule and CD, leading to complexation through spectral changes of characteristic bands of the guest molecule. 5-FU exhibits principle absorption bands at 3124 (NH), 1656–1723 cm^−1^ and 1716 (C=O), 1242 (C–O), 1300–1550 (C=C, C=N), 813 and 1245 (CH), 2400–3100 (aromatic ring), 1429 (mono-substituted pyrimidine ring) and 1100 cm^−1^ (C–F).^46–49,54^ The FTIR spectra of freeze-dried samples of 5-FU-CD (Fig. 2) showed a shift in bands from 1656 to 1723 cm^−1^ and the disappearance of bands in the region 2400–3100 cm^−1^ indicating the formation of 5-FU-CD complexes.

### 3.2 Assay of 5-FU in photolysed solutions

The assay of 5-FU in polymeric nanoparticles,^55^ pharmaceutical preparations^9^, and biological fluids^56^ has been carried out by stability-indicating HPLC methods. 5-FU and photoproducts in photodegraded solutions have been determined by HPLC-MS/MS,^17,57^ UPLC-HRMS^21^ and LC-IT-MS/MS.^16,20^ In the present study, the HPLC assay method for the determination of 5-FU in the presence and absence of CDs (α-, β-, and γ) has been developed and validated using ICH guidelines.^53^ The results obtained are given in Table 1, and the chromatograms for 5-FU in the absence and presence of CDs (α-, β-, and γ) are given in Fig. S2. The developed HPLC method is linear for the determination of 5-FU (0.5–5.0 × 10^−5^ M) in the presence of CDs (α-, β-, and γ-) (Fig. S3).

**Table 1:** Calibration data of 5-FU in the presence of CDs using the straight line equation *y* = *mx* + *c*^a^

	α-CD	β-CD	γ-CD
*λ*_ma*x*_, nm	266	266	266
Retention time (*t*_R_), min	3.40	3.40	3.40
%RSD	1.15	1.11	1.09
Theoretical plates (N)	13990	13600	14051
Tailing factor (T)	1.12	1.24	1.09

Linearity			
Range (M ×10^5^)	0.50–5.00	0.50–5.00	0.50–5.00
Correlation coefficient	0.9997	0.9998	0.9999
Slope (M × 10^10^)	1.68	2.13	2.56
SE of slope (M × 10^3^)^b^	6.26	4.90	5.78
Intercept (×10^3^)	0.40	6.39	8.48
SE of intercept (×10^3^)^b^	4.28	3.35	3.95
SD of intercept (×10^4^)^c^	1.35	0.16	1.25
Accuracy (%)±SD	100.0 ± 1.54	100.1 ± 1.24	99.65 ± 1.56
Precision (%RSD)	1.54	1.23	1.57
Robustness (mean accuracy (%))±SD (%RSD)	100.2 ± 0.36 (0.35)	101.5 ± 0.22 (0.22)	101.3 ± 0.45 (0.45)
LOD (M ×10^6^)^d^	2.65	1.64	1.61
LOQ (M ×10^6^)^e^	8.40	4.97	4.88

a where, *m* = slope and *c* = *y*-intercept.

b SE = standard error.

c SD = standard deviation.

d LOD = limit of detection.

e LOQ = limit of quantification.

### 3.3 UV spectra of 5-FU

**Fig. 3:**
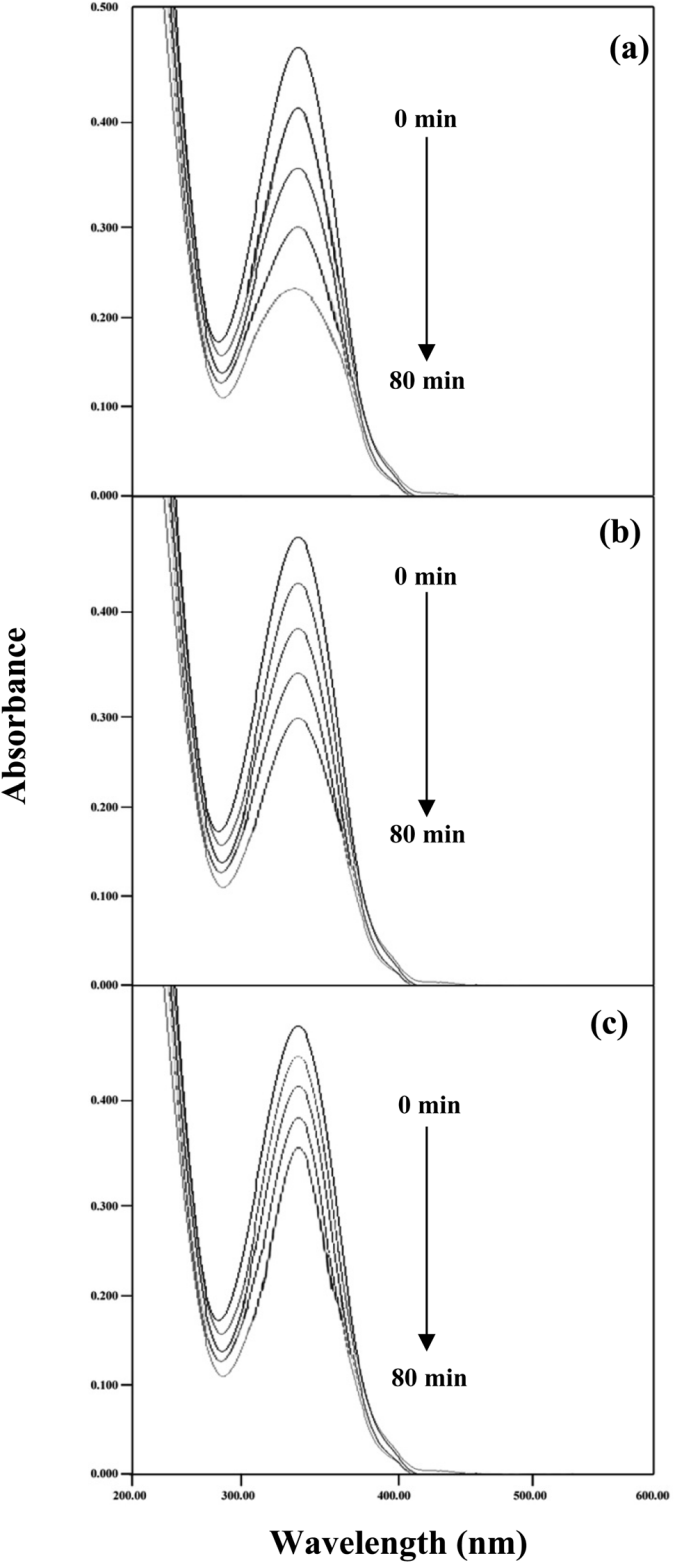
Spectral changes of 5-FU (5 × 10^−5^ M) on photolysis in the presence of CDs (1.25 × 10^−3^ M) (pH 9.0): α-CD (a), β-CD (b), and γ-CD-CD (c).

5-FU exhibits an absorption maximum in aqueous solution at 266 nm.^4,58,59^ The UV irradiation of 5-FU solutions in the presence of CDs shows a loss of absorption at 266 nm, indicating the degradation of the molecule. Typical sets of absorption spectra of 5-FU solutions (pH 9.0) obtained in the presence of CDs on UV irradiation are shown in Fig. 3. The loss of 5-FU absorbance at 266 nm is greater in the presence of α-, followed by β-, and γ-CD. On the contrary, the loss of absorbance at 266 nm is much greater on the UV irradiation of pure 5-FU solutions, suggesting that CDs act as stabilizers of 5-FU by complexation in aqueous solution.

### 3.4 Fluorescence characteristics of 5-FU

The effect of CD entrapment on the fluorescence of 5-FU has not been reported. In the present study, it has been observed that CD causes quenching of 5-FU fluorescence, and a decrease in fluorescence occurs with an increase in concentration of CDs. A percent fluorescent loss of 5-FU in the presence of CDs (0.25–1.25 × 10^−3^ M) at pH 7.0 occurs up to 53% (α-CD), 63% (β-CD), 75% (γ-CD) (Table S1). This loss in fluorescence of 5-FU on entrapment in CDs is due to complexation between the two molecules. The Stern–Volmer quenching constant (*K*_sv_) can be calculated from the fluorescence data using the Stern–Volmer equation.^60^3.0
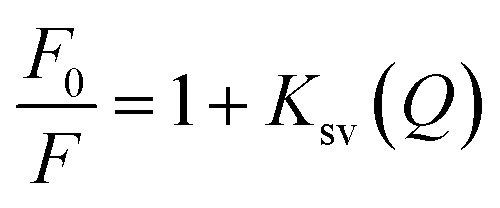
where, *F*_0_ and *F* are fluorescence intensities of 5-FU in the absence and presence of a quencher (CD), respectively. [*Q*] is the concentration of the quencher, and *K*_sv_ is called the Stern–Volmer quenching constant. The plots of *F*_0_/*F versus* CD concentration are shown in Fig. S4, and the values of *K*_sv_ for α-, β-, and γ-CDs are reported in Table 2. The *K*_sv_ values indicate that γ-CD causes the highest quenching of 5-FU.

**Table 2:** Stern–Volmer quenching constants (*K*_sv_) of 5-FU by CDs, binding constants (*K*) and number of binding sites (*n*) at 25 °C

CDs	*K*_sv_ (×10^−3^ L mol^−1^)	*K* (×10^−3^ L mol ^−1^)	*n*
α	1.10	2.89	0.94
β	1.69	3.11	0.95
γ	3.06	3.62	1.03

### 3.5 Binding constants and binding sites

The binding of a small molecule to a set of equivalent sites on a macromolecule can be expressed by the following equation.^61^4.0
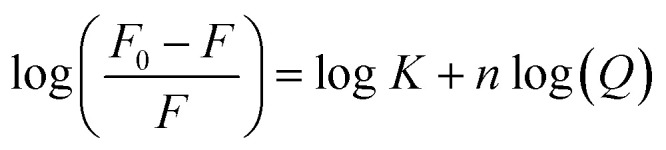
where, *K* and *n* are the binding constant to a site and the number of binding sites on the CD molecule, respectively. The values of *K* and *n* can be determined from a plot of log(*F*_0_*F*)/*F versus Q* (Fig. S5) and are reported in Table 2. These values are in order, indicating the highest binding capacity of γ-CD.

### 3.6 Entrapment efficiency of CDs

Entrapment yields of 5-FU in different CDs have been determined from the fluorescence data (Table S2) and can be used to determine the entrapment efficiency of 5-FU in different CDs as follows;5.0
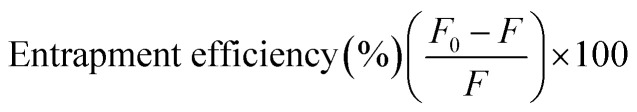
where, *F*_0_ is the fluorescence of 5-FU solution before entrapment and *F* is the fluorescence of non-entrapped 5-FU in the solution.^36^

The values of entrapment efficiencies of 5-FU in the presence of CDs (0.25–1.25 × 10^−3^ M) are reported in Table S2. The data show that 22–77% entrapment of 5-FU in CDs can be achieved. γ-CD gives the highest value (77%) compared to that of β-CD (64%) and α-CD (58%) at the highest concentration. These results show that maximum photoprotection of 5-FU can be achieved by entrapment in γ-CD under these conditions.

### 3.7 Conductivity studies

The formation of inclusion complexes of CD with different compounds has been studied by conductivity measurements since the conductivity of the ionic species depends on the concentrations of CDs.^62,63^ The technique has been used to study complexation between 1-methyl-3-octyl imidazolium tetrafluoroborate and β-CD^64^ and ascorbic acid and β-CD.^65^ It can be used to determine the stoichiometric ratio of inclusion complexes.^63^

The present study has shown that when 5-FU enters the cavity of α-, β-, and γ-CDs, the electrical conductivity of the aqueous solutions decreases with an increase in the CD concentration (α-CD < β-CD < γ-CDs) (Fig. S6). The electrical conductivity plots show a sharp curve at which the concentration of 5-FU and CDs are similar which indicates the equimolar ratio (1 : 1) of the 5-FU-CDs (2.00 mM) inclusion complexes.

### 3.8 Stoichiometry of 5-FU and CDs

The Job's method of continuous variations has been used to determine the stoichiometric complexation between 5-FU and CDs (α-, β-, and γ-). The mole fractions were continuously varied, but the total concentration of 5-FU and CDs was kept constant ([5-FU] + [CDs] = 1.00 × 10^−4^ M). A plot of change in absorbance (Δ*A*) *versus* mole fractions has been prepared and given in Fig. S7. The change in absorbance values (Δ*A*) reached its highest at 0.5 mole fraction in all CDs (α-, β-, and γ-), indicating that 5-FU forms a 1 : 1 complex with α-, β-, and γ-CDs as reported earlier for 5-FU-α/β-CD,^66,67^ betuline 3,28 dipthalate/betuline 3,28 disuccinate-γ-CD^68^ and sumanene-γ-CDs,^69^*etc.*

### 3.9 Differential scanning calorimetry (DSC)

Differential scanning calorimetry (DSC) has been used to study the thermal behavior of free 5-FU and 5-FU-CD samples, and the formation of 5-FU-CD complexes has been confirmed by this method.^46,48^ The DSC thermograms of 5-FU, 5-FU-CD, and CD (30–300 °C region) are shown in Fig. S8. 5-FU gives a sharp endothermic peak at 285 °C (Fig. S8a), which has been found to disappear in the thermograms of 5-FU-CD samples. These results show that on freeze drying of the 5-FU-CD samples, complexation has taken place. The endothermic peak at 185 °C for α-CD indicates the melting followed by its decomposition, which is due to the breakdown of its crystalline structure and leads to its thermal degradation. A broad endothermic peak appeared at 118 °C for β-CD, which is due to the release of strongly bonded water molecules present outside the cavity. These results are in agreement with the observations of previous workers on 5-FU-β-CD complexation.^46,48^ Also at 210 °C, 5-FU-β-CD shows an endothermic peak due to the release of water molecules and the decomposition of 5-FU and β-CD. 5-FU-γ-CD shows exothermic and endothermic peaks at 225 and 230 °C, respectively, which is due to the decomposition reactions (bond cleavage, oxidation) and degradation of the inclusion complex and breakdown of the hydrogen bonds of γ-CD, respectively.

### 3.10 NMR spectroscopy

**Fig. 4:**
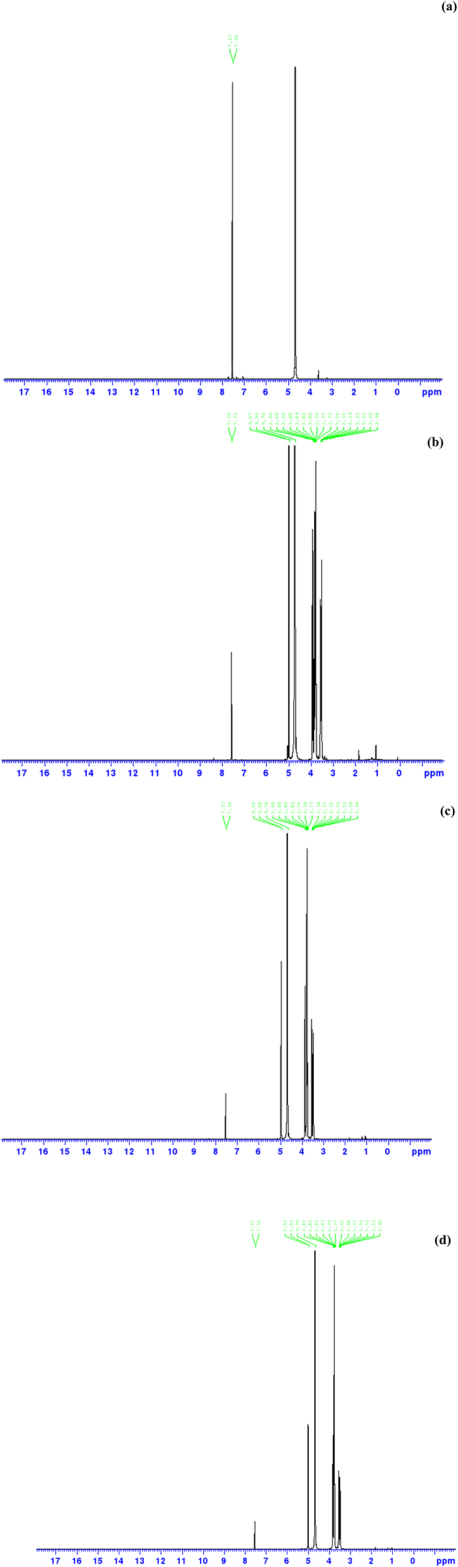
^1^H NMR spectra of 5-FU (a), 5-FU-α-CD (b), 5-FU -β-CD (c), 5-FU-γ-CD (d) in D_2_O

5-FU NMR spectrum is given in Fig. 4a, and the HDO signal was found at 4.70 ppm, whereas the doublet peak at 7.61 ppm corresponds to the CH bond. The doublet peak of CH is due to the attachment of NH adjacent to the CH bond, and spin–spin constants are reported earlier.^70,71^ The NMR spectra of 5-FU in the presence of CDs (α-, β-, γ-) are given in Fig. 4b–d. The investigation of inclusion complexation of CDs (α-, β-, γ-) with 5-FU has been evaluated using NMR spectroscopy. The details of NMR spectra obtained for 5-FU alone and in the presence of CDs (α-, β-, γ-) are given in Table S3. It has been found that there are no spectral changes or there may be some small changes that are not evident between 5-FU and CDs as reported earlier.^71^

The pure sample of 5-FU gives a doublet for H-6 at 7.61 ppm (Fig. 4a). However, the intensity of this peak of 5-FU reduced in the order of γ-CD > β-CD > α-CD (Fig. 4b–d), indicating the formation of inclusion complexes. The reduction in the intensity (α-CD < β-CD < γ-CD) shows that there is the strongest affinity of 5-FU with γ-CD followed by β-CD and α-CD. The cavity size of α-, β-, and γ-CD is 4.7–5.3 °A, 6.0–6.5 °A, and 7.5–8.3 °A, respectively.^22^ Depending on the size of the cavity, a maximum of 5-FU is found to fit in the cavity of γ-CD followed by β-CD and α-CD, indicating the strong interaction of 5-FU with γ-CD as compared to that of β-CD and α-CD which is also evident from the kinetic study, as the rate of photodegradation of 5-FU is minimum in γ-CD than β-CD and α-CD. Also, from the reduction in the intensity of the characteristic peaks of 5-FU, the encapsulation of 5-FU in CDs has been calculated, which is in the order of γ-CD (∼80%) > β-CD (∼69%) > α-CD (58%), indicating that the γ-CD possesses the strongest interaction with 5-FU as compared to that of β-CD and α-CD, which results in the maximum stability of 5-FU in the case of γ-CD followed by β-CD and α-CD (Table 5).

### 3.11 Computational analysis

**Fig. 5:**
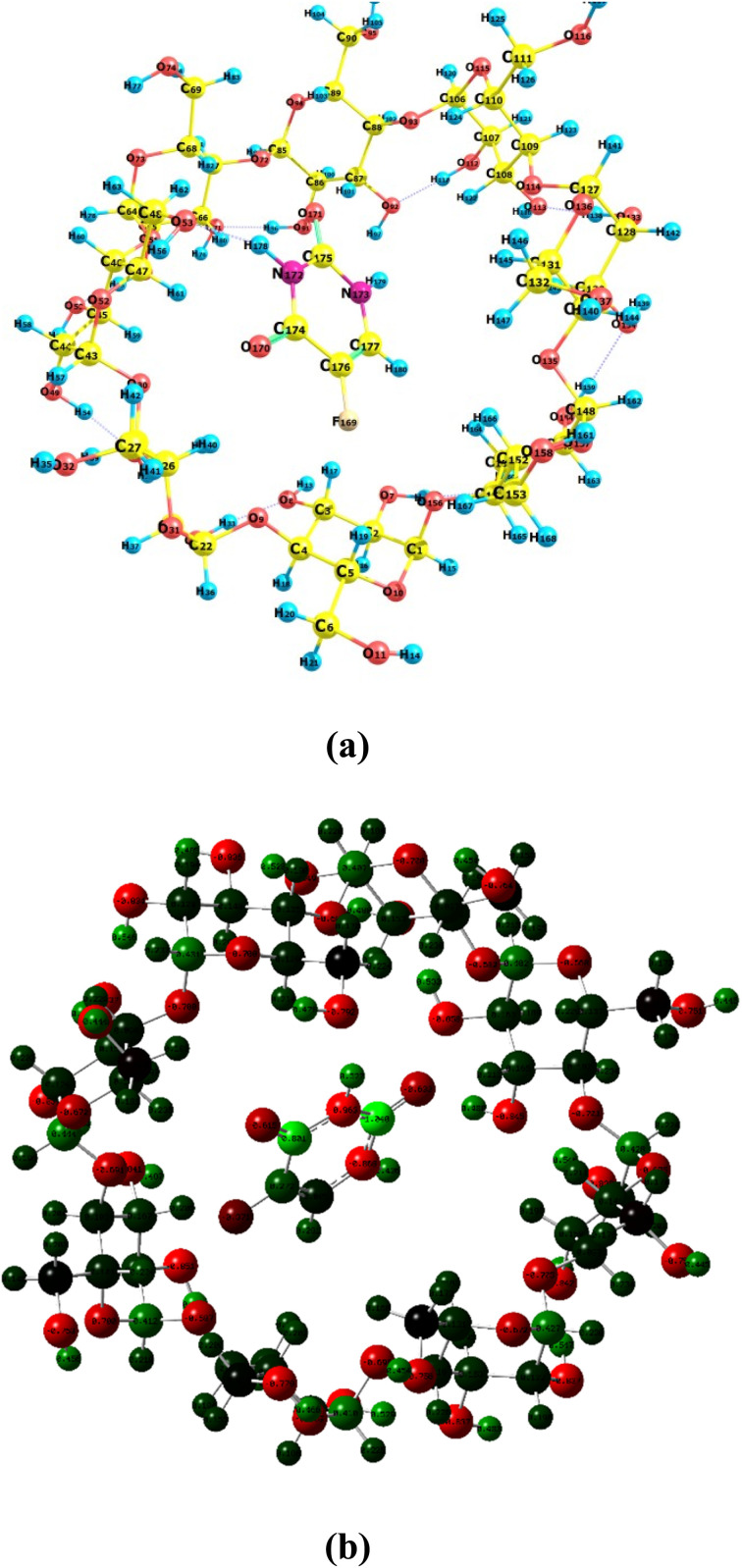
Optimized geometry of 5-FU and γ-CD complex (a) and Mulliken population analysis and pictorial representation of atoms by the charge (b), where positive charges are given in green while negative as red, based on their intensities.

The determination of characteristics of complex molecules that require simplification, the molecular geometry, plays an essential role in scientific analysis. An insightful framework for the chemical realm, having application in both early and latter stages of analysis, is gained by conducting molecular modelling studies. The optimized structures of the studied 5-FU-γ-CD complex were visualized using Avogadro and are given in Fig. 5a along with their atomic numbering.

#### 3.11.1 Mullekin charge analysis

**Fig. 6:**
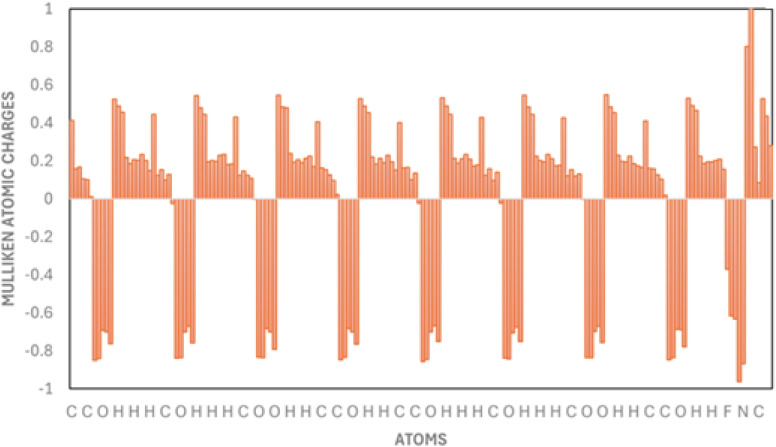
Histogram of Mulliken atomic charges for 5-FU and γ-CD complex.

The electronic structure, polarizability, and properties of molecules are largely affected by the atomic charges. Therefore, the Mullekin population analysis plays an important role in quantum chemical operations. Mullekin atomic charges for 5-FU complexed with γ-CDs were computed through the Hartree–Fock (HF) method with the 6-31G(d) basis set and are represented as a histogram in Fig. 6. The Mulliken atomic charges showed the existence of electrophilic sites as well as nucleophilic sites. The carbon atoms having charge ∼+0.59 to +0.61 show moderately electron-deficient centers, the oxygen atoms with charges ∼−0.48 to −0.59 depict electron rich centers, being potential hydrogen bond acceptors, the F169: −0.35 is highly electronegative and electron rich center, the N172 and N173: +0.07 to +0.15 show miildy positive nature having potential hydrogen bond donor characteristics. Some hydrogens are typically positive while some are negative due to the bonding environment. The Mulliken charge distribution for the equilibrium geometry of γ-CD complexed with 5-FU is given in Fig. 5b, where the positive charges are given in green and the negative charges in red, based on their intensities.

The moderate positive charge on nitrogen and fluorine atoms of 5-FU indicates they might not be highly nucleophilic when complexed. Some hydrogen atoms inside the CD cavity, shown green, suggest polarization due to interaction with 5-FU. The high electron density on the oxygen atoms of γ-CD, specifically the hydroxyl groups on the rims of cyclodextrin, which appears red, shows hydrogen bonding with hydrogens on 5-FU. The carbonyl oxygen and possibly the fluorine within the 5-FU carry negative charge, suggesting potential sites for interaction with the more positive regions of the CD's cavity. Possible hydrogen bonding, dipole–dipole interactions, or electrostatic stabilization could be there based on differential charge distribution between 5-FU and the OH in the CDs ring. Encapsulation of 5-FU by the CD cavity led to shielding of electron-rich reactive sites, that is, carbonyl oxygen and fluorine atom, reduction in the photochemical reactions such as electron excitation or radical formation, which are typically initiated at these nucleophilic sites. This enhanced redistribution upon complexation could be the reason for the enhanced photostability of 5-FU.

#### 3.11.2 Natural bond orbital (NBO) analysis

NBO analysis is an efficient method to study the intra- and inter-molecular bonding and to understand the charge transfer or conjugative interactions in a molecular system. The NBO analysis was performed on the molecule at HF/6-13 G(d) level to understand the intermolecular interaction and delocalization of electron density within the molecule. Out of 754 electrons, 99.13% of electrons are in Lewis-type (bonding or lone pair) orbitals, and only 0.87% are non-Lewis, which are mainly delocalized or antibonding electrons. The Lewis structure depicts 97 core electrons corresponding to inner-shell (1s) electrons, the 192 bonding (BD) orbitals representing covalent bonds, and 88 lone pairs mostly on electronegative atoms like oxygen, nitrogen, and fluorine, common in both 5-FU and γ-CD. A minor deviation from perfect 2-electron core orbitals is observed, which might be due to polarization by nearby electronegative atoms like fluorine or oxygen on 5-FU, and hyperconjugation or minor delocalization into Rydberg or antibonding orbitals. This suggests some carbon atoms have slightly delocalized core orbitals due to electronic environment asymmetry in the complex because of inclusion in the γ-CD cavity. The analysis also confirmed delocalized π-systems due to the uracil ring in 5-FU. The NBO structure also showed a resonance hybrid and not a single Lewis structure, implying potential charge delocalization between the guest (5-FU) and the host (γ-CD) through weak orbital overlap or hydrogen bonding. The Lewis occupancy across the cycle was observed to be stable (∼747–747.4), while the non-Lewis occupancy, ∼6.55–6.79, showed low deviation, indicating no convergence problems or electronic instability (Table 3). At more relaxed thresholds, that is, at 1.50, non-Lewis contributions increase, uncovering more delocalization or hyperconjugative effects likely due to hydrogen bonding between the γ-CD hydroxyls and the 5-FU carbonyl or nitrogen atoms, indicating possible π–π or n → π* interactions within the cavity. The sight core orbital deviations on specific carbon atoms (C2, C23, C86) may be due to fluorine atom electron withdrawing effects in 5-FU and the asymmetric inclusion into γ-CD, changing local fields.

**Table 3:** Natural bond orbital analysis of complex 5-FU and γ-CD

Cycle	Occ. Thresh.	Occupancies		Lewis's structure				Low occ. (L)	High occ (NL)	Dev
		Lewis	Non-Lewis	CR^a^	BD^b^	3C^c^	LP^d^			
1 (1)	1.90	747.20565	6.79435	97	192	0	88	4	4	0.11
2 (2)	1.90	747.43958	6.56042	97	191	0	89	3	3	0.11
3 (3)	1.90	747.20565	6.79435	97	192	0	88	4	4	0.11
4(4)	1.90	747.20565	6.79435	97	192	0	88	4	4	0.11
5(5)	1.90	747.20565	6.79435	97	192	0	88	4	4	0.11
6 (6)	1.90	747.20565	6.79435	97	192	0	88	4	4	0.11
7 (7)	1.90	747.20565	6.79435	97	192	0	88	4	4	0.11
8 (8)	1.90	747.20565	6.79435	97	192	0	88	4	4	0.11
9 (9)	1.90	747.20565	6.79435	97	192	0	88	4	4	0.11
10 (1)	1.80	747.17157	6.82843	97	191	0	89	2	3	0.60
11 (2)	1.80	747.44421	6.55579	97	191	0	89	2	3	0.11
12 (3)	1.80	747.17157	6.82843	97	191	0	89	2	3	0.60
13 (4)	1.80	747.44421	6.55579	97	191	0	89	2	3	0.11
14 (1)	1.70	747.44428	6.55572	97	191	0	89	1	3	0.11
15 (2)	1.70	747.17165	6.82835	97	191	0	89	1	3	0.60
16(3)	1.70	747.44428	6.55572	97	191	0	89	1	3	0.11
17(1)	1.60	747.44489	6.55511	97	191	0	89	0	3	0.11
18(2)	1.60	747.44489	6.55511	97	191	0	89	0	3	0.11
19(1)	1.50	736.76745	17.23255	97	170	0	110	1	24	0.92
20(2)	1.50	738.04436	15.95564	97	170	0	110	0	22	0.73
21(3)	1.50	738.04436	15.95564	97	170	0	110	0	22	0.73
22 (1)	1.60	747.44489	6.55511	97	191	0	89	0	3	0.11

a CR = Central atom (possibly context-specific).

b BD = Bonding electrons.

c 3C = Three-center bond (in some special molecules).

d LP = Lone pair of electrons.

The perturbation energies of donors and acceptors are given in Table 4. A large amount of data was obtained; however, the top ten interactions with the greatest value of stabilization energy are shown. A strong LP (O) → BD(C = O)* or *BD(C–N)** indicates n → π* interactions that stabilize the π* orbital of 5-FU and is shown to be enhanced when hydrogen bonded to γ-CD hydroxyls. The lone pair on oxygen 112 is donating into the π* antibonding orbital of carbonyl 113–114, causing strong delocalization or hydrogen bond reinforcement. Hyperconjugating stabilization is also observed, in which some interactions involve bonding orbitals donating into neighbouring antibonding orbitals. This phenomenon is common in ring γ-CD, which helps to maintain the ring flexibility and stabilization. Several interactions occurring between lone pairs on γ-CD oxygen atoms and antibonding orbitals of 5-FU or sugar C–C bond having high E(2) values indicated significant stabilization due to hydrogen bonding, hyperconjugation, or π-electron delocalization effect. The NBO analysis revealed the redistribution of electron density after encapsulation of 5-FU with γ-CD, indicating reduced susceptibility to photoinduced electron excitation, a major cause of photodegradation, mainly due to the electron withdrawal from photoreactive centers. The donor–acceptor interaction between LP(oxygen of γ-CD) → π* (carbonyl of 5-FU) stabilizes the π* orbitals and lowers their energy, making them less accessible to photoexcitation. Moreover, the strong hydrogen bonding between LP → σ*/π* interactions more likely restricted the molecular motion of 5-FU inside γ-CD cavity, minimized conformational changes or torsional relaxation after UV absorption, and acted as a physical and electronic shield preventing access to UV photons. The higher *E*^2^ value also suggested increased delocalization of electron density, decreasing the likelihood of forming excited state radicals or reactive intermediates, which are common pathways for the photodegradation.

**Table 4:** Second-order perturbation theory analysis of the Fock matrix in NBO basis in 5-FU and γ-CD complex

Donor (i)	Type	ED (e)	Acceptor (j)	Type	ED (e)	E(2)^a^ [kJ mol^−1^]	E(j)–E(i)^b^ [A.U.]	F(i, j)^c^ [A.U.]
LP(3) O112	LP	1.8612	BD*(2) C113–O114	π*	0.3861	24.64	0.66	0.084
LP(2) O112	LP	1.9780	BD*(2) C113–O114	π*	0.3861	23.64	0.61	0.078
LP(2) O78	LP	1.9779	BD*(2) C79–O80	π*	0.3845	19.55	0.62	0.070
LP(2) O5	LP	1.9766	BD*(2) C3–C4	σ*	0.0715	17.19	0.48	0.063
LP(2) O143	LP	1.9710	BD*(2) C142–O144	π*	0.3852	16.31	0.65	0.062
LP(2) O140	LP	1.9643	BD*(2) C142–O144	π*	0.3852	15.40	0.59	0.061
LP(2) O18	LP	1.9708	BD*(2) C16–C17	σ*	0.0733	14.27	0.53	0.059
LP(2) O64	LP	1.9711	BD*(2) C63–C65	σ*	0.0727	13.47	0.54	0.057
LP(2) O47	LP	1.9724	BD*(2) C46–O48	π*	0.3873	13.18	0.58	0.055
LP(2) O39	LP	1.9725	BD*(2) C37–C38	σ*	0.0730	12.10	0.50	0.052

a Second-order perturbation energy.

b Energy difference between acceptor (*E*_j_) and donor (*E*_i_) orbitals.

c Fock matrix element.

#### 3.11.3 Molecular docking

**Fig. 7:**
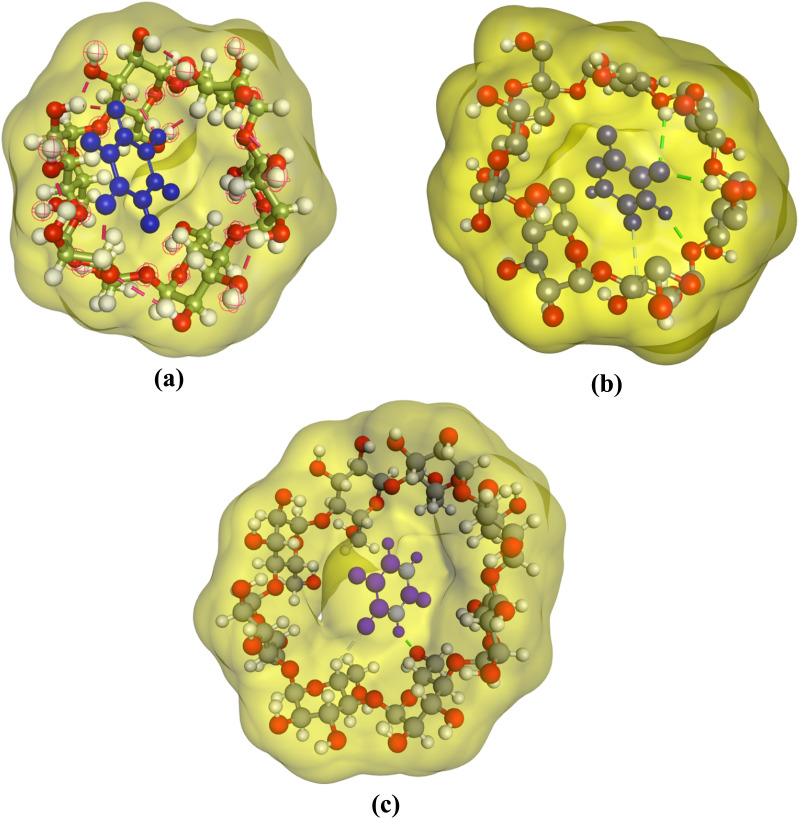
3D illustration of 5-FU inclusion complexes using molecular docking: (a) 5-FU–α-CD (b) 5-FU-β-CD, (c) 5-FU-γ-CD.

The inclusion complexes formed due to the host–guest relationship between CDs (α-, β-, γ-) and 5-FU have been evaluated using molecular docking simulations (MDS) studies. MDS studies are useful software for the evaluation of the stability and inclusion of complex formations between two or more chemical compounds having known structures. The inclusion complexes formed between 5-FU and CDs (α-, β-, γ-) have been generated using BIOVIA Discovery Studio Visualizer. The 3D structures of CDs and 5-FU were downloaded from the protein data bank (PDB) and PubChem, respectively. The simulations obtained agreed with results obtained from conductometry, UV spectroscopy, FTIR, and DSC, indicating that 5-FU forms a 1 : 1 inclusion complex with CDs (α-, β-, γ-). The molecular docking model obtained for 5-FU and CDs is given in Fig. 7. It has been found that 5-FU is a guest molecule entering the hydrophobic cavity of α-, β- and γ-CDs (Fig. 7). The binding energies obtained are negative for 5-FU-α-CD, 5-FU-β-CD, and 5-FU-γ-CD inclusion complexes are −3.60, −4.10, and −3.80, respectively, indicating the stability and binding affinity of 5-FU inclusion complexes formed with CDs (α-, β-, γ-).

### 3.12 Kinetics of photostabilization of 5-FU in the presence of CDs

#### 3.12.1 Rate constants for photodegradation of 5-FU

The kinetics of photodegradation of 5-FU alone and on entrapment in CDs in aqueous solution have been studied. 5-FU has been found to undergo photodegradation by first-order kinetics. In the present study, the photodegradation of 5-FU by UV light (254 nm, closely corresponding to the absorption maximum of 5-FU at 266 nm)^59^ has been carried out at pH 2.0–12.0. The reaction follows first-order kinetics, and the values of first-order rate constants in the absence (*k*_0_) and presence of CDs (*k*_obs_) are reported in Table 5. The values of *k*_obs_ for 5-FU in the presence of α-, β-, and γ-CDs are lower than those of the values of *k*_0_ (in the absence of CD). Moreover, the values of *k*_obs_ have been found to decrease in the order: α-CD > β-CD > γ-CD, indicating the effect of CDs on the inhibition of the rate of photodegradation and hence the photostabilization of 5-FU. The first-order rate constants (*k*_obs_) are found to be in the range of 2.25–5.12, 2.19–4.89, and 2.08–4.69 × 10^−3^, min^−1^ in the presence of α-, β-, and γ-CD, respectively (Table 5).

**Table 5:** First-order rate constants for photodegradation of 5-FU (*k*_obs_), in the presence of α-, β- and γ-CDs and quantum yields (*Φ*) of the reaction^a^

pH	CD Concentration (M × 10^3^)	*k*_obs_ × 10^3^, min^−1^			*k*_0_ × 10^3^, min^−1^	*Φ*
		α-CD	β-CD	γ-CD		
2.0	0.25	3.38	3.30	3.19	3.51	—
	0.50	3.26	3.15	2.85		—
	0.75	3.15	2.96	2.49		—
	1.00	3.01	2.85	2.16		—
	1.25	2.89	2.64	1.85		0.065
3.0	0.25	3.01	2.89	2.75	3.10	—
	0.50	2.89	2.72	2.42		—
	0.75	2.76	2.50	2.15		—
	1.00	2.64	2.29	1.87		—
	1.25	2.56	2.10	1.50		0.053
4.0	0.25	2.50	2.48	2.31	2.61	—
	0.50	2.41	2.26	2.06		—
	0.75	2.29	2.17	1.75		—
	1.00	2.19	1.97	1.49		—
	1.25	2.10	1.80	1.14		0.040
5.0	0.25	2.25	2.19	2.09	2.36	—
	0.50	2.15	2.01	1.75		—
	0.75	2.04	1.85	1.48		—
	1.00	1.94	1.69	1.16		—
	1.25	1.86	1.54	0.92		0.032
6.0	0.25	2.03	1.92	1.89	2.11	—
	0.50	1.95	1.75	1.67		—
	0.75	1.84	1.56	1.36		—
	1.00	1.80	1.40	1.15		—
	1.25	1.75	1.21	0.85		0.030
7.0	0.25	2.12	2.04	1.98	2.22	—
	0.50	2.05	1.85	1.75		—
	0.75	1.98	1.72	1.50		—
	1.00	1.89	1.59	1.32		—
	1.25	1.82	1.38	1.05		0.037
8.0	0.25	2.34	2.24	2.17	2.45	—
	0.50	2.27	2.11	1.96		—
	0.75	2.13	1.92	1.72		—
	1.00	2.06	1.72	1.49		—
	1.25	1.94	1.57	1.22		0.043
9.0	0.25	2.49	2.45	2.39	2.59	—
	0.50	2.38	2.28	2.17		—
	0.75	2.29	2.09	1.89		—
	1.00	2.13	1.95	1.65		—
	1.25	2.04	1.80	1.51		0.053
10.0	0.25	2.69	2.60	2.52	2.78	—
	0.50	2.61	2.45	2.39		—
	0.75	2.51	2.31	2.09		—
	1.00	2.44	2.09	1.99		—
	1.25	2.36	1.98	1.73		0.061
11.0	0.25	3.74	3.69	3.54	3.81	—
	0.50	3.68	3.48	3.26		—
	0.75	3.62	3.27	2.89		—
	1.00	3.56	3.06	2.50		—
	1.25	3.49	2.85	2.21		0.078
12.0	0.25	5.12	4.89	4.69	5.22	—
	0.50	5.03	4.52	4.26		—
	0.75	4.96	4.06	3.69		—
	1.00	4.88	3.69	3.06		—
	1.25	4.81	3.47	2.62		0.095

a Quantum yield of photolysis in the presence of γ-CD.

The formation of 5-FU-CD inclusion complexes is well established.^71^ In order to examine the interaction of 5-FU and CDs, photodegradation of 5-FU was carried out in the presence of 0.25–1.25 × 10^−3^ M concentrations of α-, β-, and γ-CDs, and the values of *k*_obs_ were determined (Table 5). The second-order rate constants, *k*_2,_ for the photochemical interactions of 5-FU and CDs have been obtained from the slopes of linear curves (Fig. S9) and are reported in Table 6. The values of these rate constants vary with pH and indicate that the 5-FU-CD interaction is greater in the presence of γ-CD compared to that of α- and β-CD, causing greater stabilization of 5-FU. The second-order rate constants (*k*_2_) are found to be in the range of 0.25–0.46, 0.63–1.47, and 0.79–2.14, M^−1^, min^−1^ for α-, β-, and γ-CD, respectively (Table 6), indicating that higher photochemical interaction between γ-CD followed by β-CD and α-CD results in the increase in the stability of 5-FU in the order of γ-, β- and α-CD.

**Table 6:** Second-order rate constants (*k*_2_) for photochemical interaction of 5-FU with α-, β- and γ-CDs at pH 2.0–12.0

	*k*_2_ (M^−1^ min^−1^)		
pH	α-CD	β-CD	γ-CD
2.0	0.49	0.65	1.35
3.0	0.46	0.80	1.22
4.0	0.41	0.66	1.16
5.0	0.40	0.65	1.17
6.0	0.28	0.71	1.04
7.0	0.30	0.63	0.92
8.0	0.40	0.69	0.95
9.0	0.46	0.65	0.91
10.0	0.33	0.64	0.79
11.0	0.25	0.84	1.37
12.0	0.31	1.47	2.14

#### 3.12.2 *k*_obs_-pH profile

**Fig. 8:**
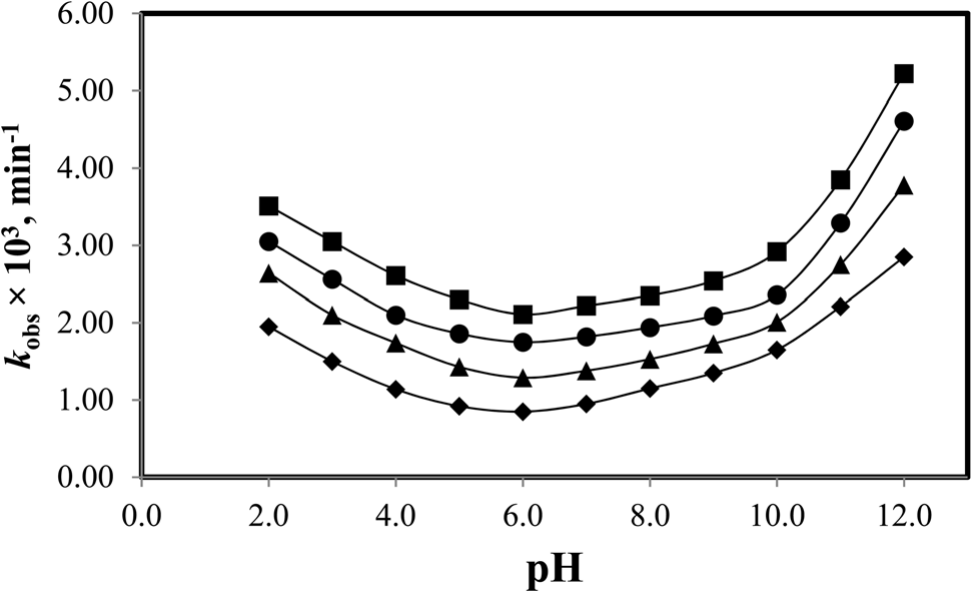
*k*_obs_-pH profiles for the photolysis of 5-FU in the absence (■) and presence of CDs: α-CD (●), β-CD (▲), and γ-CD (♦).

Stability studies are carried out to evaluate the stability of drugs at a particular pH or in the presence of other compounds (*i.e.*, caffeine, CDs). CDs are known to form inclusion and exclusion complexes with drugs (*i.e.*, indomethacin, riboflavin, metronidazole, diazepam, *etc.*), which increases the solubility and stability of drugs. 5-FU exhibits neutral (N1–H intact) and anionic (N1-deprotonated) forms at pH < 7.0 and > 8.0, respectively. However, 5-FU does not possess a basic amino group; therefore, it does not form cationic species. In order to observe the effect of pH on the photodegradation of 5-FU in the presence of CDs, the values of *k*_obs_ were plotted against pH. The *k*_obs_-pH profiles for 5-FU in the presence of CD (Fig. 8) represent U-shaped curves indicative of its general acid–base catalyzed reactions in buffered solutions. The rate is enhanced below pH 6 and above pH 10 due to the buffer ion-catalyzed reaction in the pH range 6–10, there is a near pH-independent region involving the reaction of water molecules with 5-FU. The U-shaped rate-pH profiles have been reported in the case of acid–base catalyzed degradation of cephalosporins (*e.g.*, cefotaxime and cephalothin),^72,73^ cyclophosphamide,^74^ indomethacin,^75^ oxazepam,^76^ sulfacetamide,^77^ and photodegradation of cyanocobalamin,^78^ and piroxicam.^79^ It has been found that the rate of photodegradation of 5-FU in the absence and presence of CDs shows a U-shaped curve with a maximum rate of photodegradation at pH 2.0 in the acidic region and at pH 12.0 in the alkaline region. This indicates that the neutral and anionic species of 5-FU are more susceptible to photodegradation in the absence and presence of CDs. At pH 12.0 (p*K*_a_ 8.0), the rate of photodegradation of 5-FU is maximum, where the anionic species are around 99.99%, indicating that the anionic species of 5-FU are highly susceptible to photodegradation in the absence and presence of CDs. However, at pH 2.0, the rate of photodegradation is also high, where the cationic species are around 0.0001% and the neutral species are around 99.99%, indicating that they are also highly susceptible to photodegradation. This indicates that the neutral and anionic species are both prone to photodegradation in the absence and presence of CDs. The rates of photodegradation in the absence and presence of CDs are minimal at pH 6.0, and this pH can be used to prepare photostable formulations of 5-FU alone or in the presence of CDs.

### 3.13 Quantum yields of photodegradation of 5-FU

The quantum yields (*Φ*) for the photodegradation of 5-FU at pH 2.0–12.0 in the presence of γ-CD have been determined and vary from 0.03 (pH 6.0)–0.095 (pH 12.0), compared to those of 5-FU alone (Table 5). The values of quantum yields increase with the ionization of 5-FU in the alkaline region. The higher and lower values of *Φ* indicate the reactivity of the excited-state complexes of 5-FU and γ-CD depending on the pH of the medium.

### 3.14 Mode of 5-FU-CD interaction and 5-FU photostabilization

The photooxidation of 5-FU in aqueous solution on UV irradiation results in the formation of hydroxyl derivatives by deflourination,^17,21,80^ pyrimidine ring cleavage products,^16,20,21,57^ and mineralization products.^16,17,20,81^ The deflourination of 5-FU is analogous to the main photoreaction of 6-monosubstituted fluoroquinolones, followed by hydroxylation.^82^ The photochemical interaction of 5-FU and CD has not so far been studied. A scheme for these reactions is proposed as follows.

#### 3.14.1 Photodegradation of 5-FU



6.0

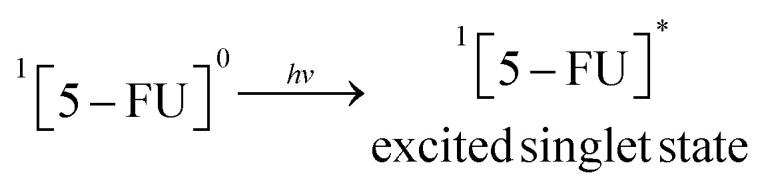



7.0

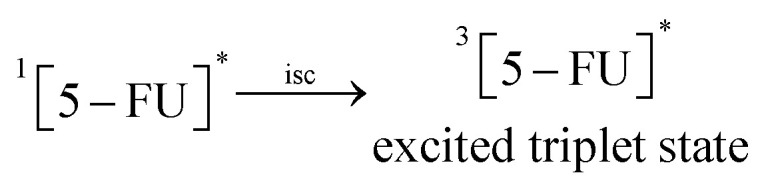



8.0

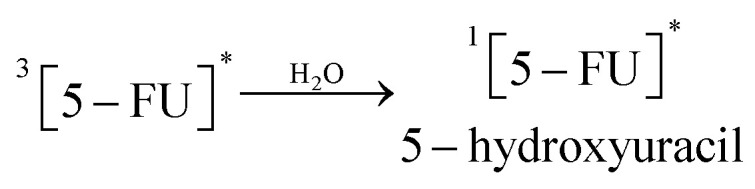



9.0

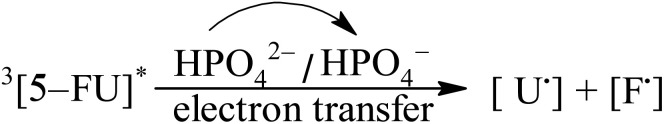

10.0 *U*
^*^ → products

^1^[5-FU]^0^ on the absorption of a photon of light is promoted to the excited singlet state (6.0), which is transformed to the excited triplet state on inter-system crossing (7.0) ^3^[5-FU]^*^. The triplet state undergoes C–F bond cleavage and the addition of a molecule of water to yield 5-hydroxyuracil and fluoride anion (8.0) as observed in the case of aromatic photosubstituted reactions through the triplet state.^83^ In the presence of buffer solutions (HPO_4_^2−^/HPO_4_^−^), the triplet state is quenched by the buffer salts, leading to electron transfer induced deflourination to form uracil and fluoride radicals (9.0) as suggested in the case of 6-monofloroquinolones.^84,85^ The uracil radical may lead to the formation of photoproducts (10.0).

#### 3.14.2 Photostabilization of 5-FU by cyclodextrins



11.0

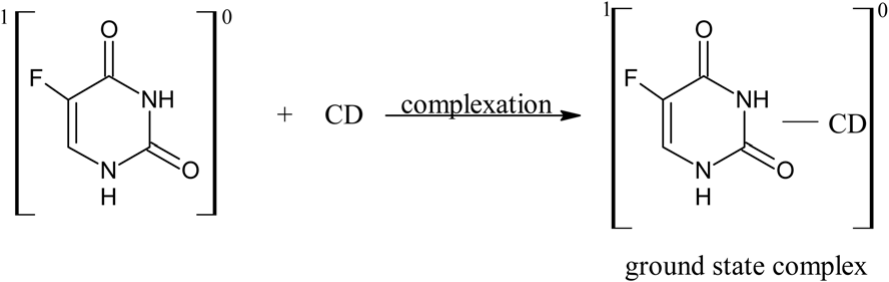



12.0

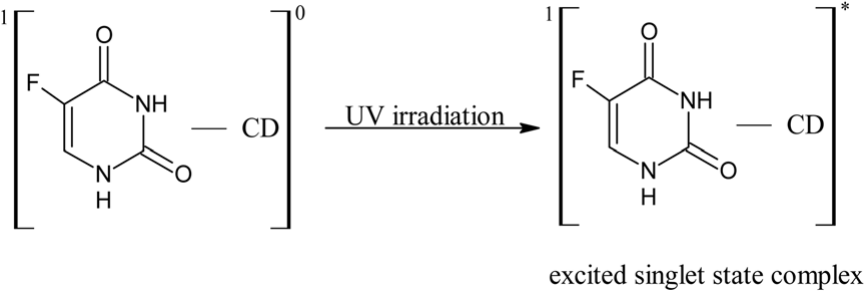



13.0

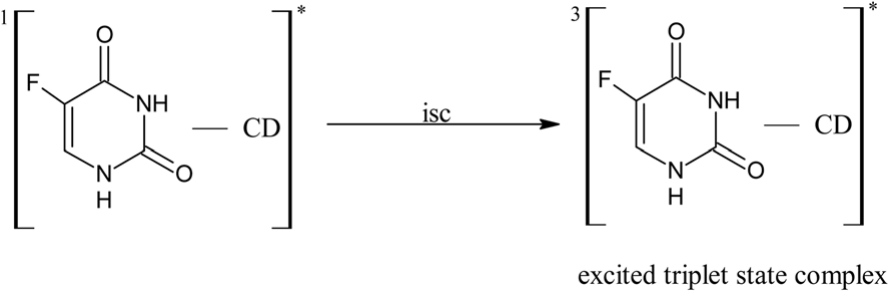



14.0

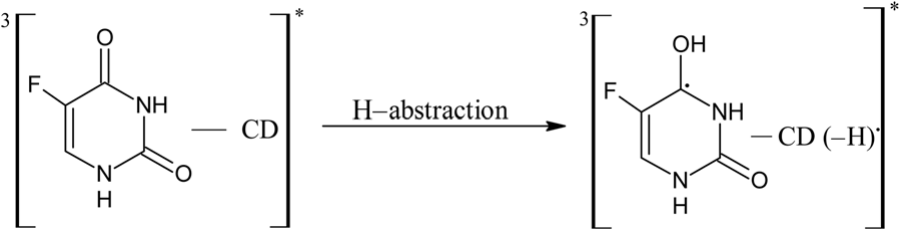



15.0

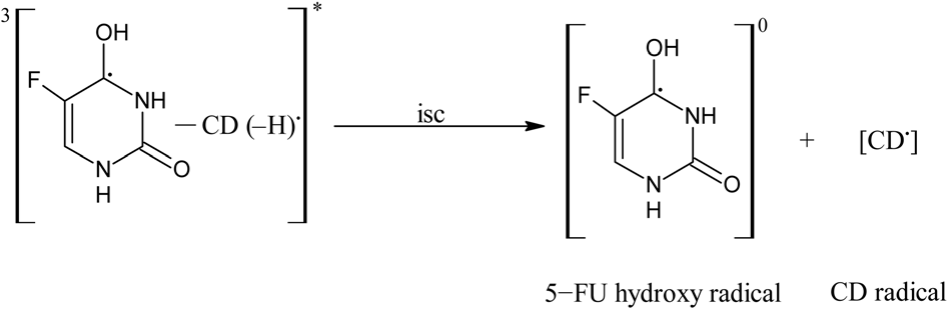



16.0

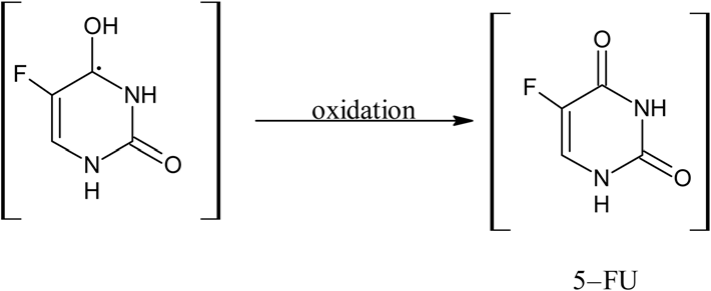



17.0

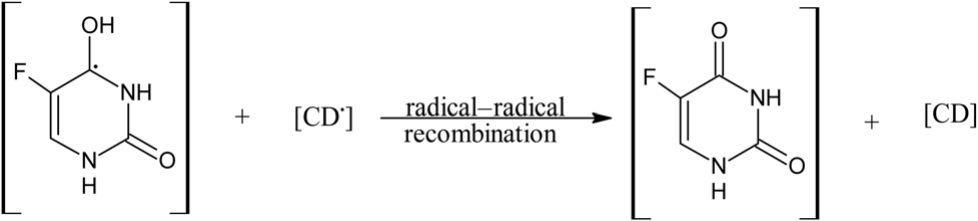

A 5-FU molecule reacts with CD to form a ground state complex (11.0), which on UV irradiation gives rise to the excited singlet state complex (12.0). This on intersystem crossing (isc) is converted to the excited triplet state complex (13.0). In the triplet state, a hydrogen-abstraction process by 5-FU takes (14.0) place to form a 5-hydroxy FU radical in association with a CD radical (15.0). The hydrogen-atom abstraction processes are common in flavins on interaction with many substrates^86–91^ and other drugs on interaction with CD in the excited state have been studied by laser flash photolysis.^92–94^ The formation of CD radicals has been observed in the photolysis of naphthoquinones^36^ and carbohydrate moieties.^95^ The 5-FU hydroxy radical on oxidation results in the formation of 5-FU (16.0). This radical pair then undergoes radical–radical recombination to yield a 5-FU and a CD molecule (17.0). Such radical–radical recombination reactions have been reported in the photodegradation of ketoprofen-β-CD complex^28,44^ tolmetin-β-CD complex,^45^ as well as tiaprofenic acid-β-CD complex^84^ and α-alkyl dibenzyl ketone-β-CD complexes. The above-mentioned scheme provides a rationale for the photochemical interaction of 5-FU and CD, resulting in the stabilization of 5-FU.

## 4. Conclusions

The present study indicates the photostabilization of 5-fluorouracil (5-FU) in the presence of cyclodextrins (CDs) (α-, β-, γ-) and finds it to be in the order of γ-CD > β-CD > α-CD. The entrapment efficiency of 5-FU is high in the γ-CD, followed by β- and α-CD. The fluorescence quenching and binding constants of 5-FU in the presence of CDs have been determined. The apparent first-order rate constants (*k*_obs_) are the maximum in the presence of α-CD, followed by β- and γ-CD, indicating that the γ-CD has maximum efficiency to stabilize 5-FU as compared to that of β- and α-CDs. The computational analysis complemented the experimental findings, providing insight into the electronic stabilization mechanism at the molecular level. The results revealed how the second-order donor–acceptor interaction led to electron delocalization and orbital stabilization, thereby reducing the availability of reactive sites for photochemical excitation. A specific HPLC assay method is a prerequisite for the correct estimation of 5-FU in photodegraded solutions. The photostabilization of 5-FU in the presence of CDs is due to the H-abstraction from CD to 5-FU, resulting in the formation of 5-fluorouracil hydroxyl radical and CD radical, which, on radical–radical recombination, results in the photostabilization of 5-FU. The computational analysis reinforces this mechanism by showing enhanced electronic coupling and stabilization within the 5-FU and γ-CD complex.

## Conflicts of interest

The authors declare that there is no conflict of interest.

## Supplementary Material

RA-015-D5RA05287D-s001

## Data Availability

Data will be available upon reasonable request to the corresponding author. The supplementary data present additional data associated with this study for readers to read. See DOI: https://doi.org/10.1039/d5ra05287d.
