# Supplementary material for: Cyclodextrin-assisted photostabilization of 5-fluorouracil: a combined kinetic and computational investigation
Source: RSC Adv. 2025 Sep 1;15(38):31194–209. doi: 10.1039/d5ra05287d (PMC12400308; doi:10.1039/d5ra05287d)
Supplement: RA-015-D5RA05287D-s001 [file RA-015-D5RA05287D-s001.pdf]

Supplementary Information (SI) for RSC Advances.
This journal is © The Royal Society of Chemistry 2025

# SUPPLEMENTARY DATA

***Research Article***

**Cyclodextrin-Assisted Photostabilization of 5-Fluorouracil: A Combined Kinetic and Computational Investigation**

Adeela Khurshid[1Ɨ], Zubair Anwar[2Ɨ*], Muneeba Usmani[1], Reem Altaf[3], Ayesha Awan[4], Zuneera Akram[5], Sadia Hafeez Kazi[1], Sofia Ahmed[1], Muhammad Ali Sheraz[1], Iqbal Ahmad[2]

[1]Department of Pharmaceutics, Baqai Institute of Pharmaceutical Sciences, Baqai Medical University, Super Highway, Gadap Road, Karachi, Pakistan

[2]Department of Pharmaceutical Chemistry, Baqai Institute of Pharmaceutical Sciences, Baqai Medical University, Super Highway, Gadap Road, Karachi, Pakistan

[3] Department of Pharmaceutical Chemistry, Faculty of Pharmacy, Capital University of Science and Technology, Islamabad Expressway, Kahuta Road, Zone-V, Islamabad.

[4]Department of Pharmacognosy, Faculty of Pharmaceutical Sciences, Riphah International University, G-7/4, 7th Avenue, Islamabad

[5]Department of Pharmacology, Baqai Institute of Pharmaceutical Sciences, Baqai Medical University, Super Highway, Gadap Road, Karachi, Pakistan

[Ɨ]Shared First Author

[*]To whom correspondence should be addressed (email: zubair_ana@hotmail.com/zubair_ana@baqai.edu.pk).

## 1. Method Validation

The high-performance liquid chromatographic (HPLC) method for the determination of 5–FU was developed and validated in the presence of α–, β–, γ–CDs ($1.25 \times 10^{-3}$ M) using the International Council on Harmonization guidelines (2005). The different parameters used in this study for the validation of 5–FU are described below.

### 1.1 System Suitability

Before analysis, the system suitability was studied by injecting 6 solutions of 5–FU at a concentration of $5 \times 10^{-5}$ M. Parameters such as retention time ($t_R$), peak area reproducibility, peak symmetry and theoretical plates were calculated.

### 1.2 Linearity and Range

The linearity of the proposed method was determined by plotting calibration curve peak area (A.U) versus concentration of 5–FU ($0.5–5.0 \times 10^{-5}$ M). The statistical parameters were calculated such as the correlation coefficient ($R^2$), slope, standard error (SE) of slope, intercept, standard error (SE) and standard deviation (SD) of slope using the straight line equation ($y = mx + c$).

where

m = slope

c = intercept

The range for the assay of 5–FU was selected from the results obtained from linearity. The studied concentration range shows linear pattern in the concentrations range.

## 1.3 Accuracy

The accuracy of the proposed method was evaluated using three different concentrations of 5–FU (1.0, 2.0, 5.0 × $10^{-5}$ M). All the solutions were made in triplicate and analyzed by HPLC. The accuracy of the proposed method was determined.

## 1.4 Precision

The precision of the proposed HPLC method was evaluated by estimating repeatability (intra–day) and intermediate (inter–day) precision. Repeatability was determined by preparing six samples of 5–FU at a concentration of 2.0 × $10^{-5}$ M. Intermediate precision was determined by different analysts on different days. The precision (%RSD) of the test method was calculated.

## 1.5 Limit of Detection (LOD) and Limit of Quantification

LOD and LOQ for the proposed method were calculated by linearity data using the formulas given below:

$$LOD = 3.3 \times \frac{\sigma}{S}$$

$$LOQ = 10 \times \frac{\sigma}{S}$$

where

$\sigma$ = standard deviation of the y–intercept

S = slope of the calibration curve

## 1.6 Robustness

Robustness of the suggested HPLC method for the determination of 5–FU was measured by making deliberate changes, i.e. wavelength (±1 nm), flow rate (±0.1 ml) and pH (±0.1 unit). The accuracy and precision of the method after these deliberate changes were determined.

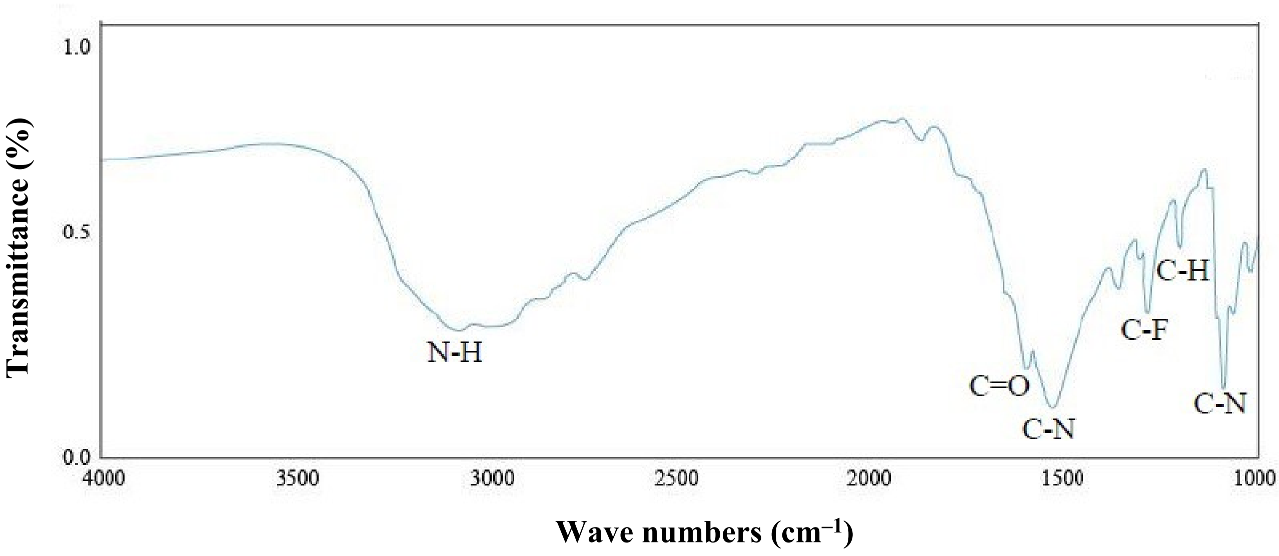


**Fig S1.** FTIR spectrum of 5–fluorouracil (5-FU)

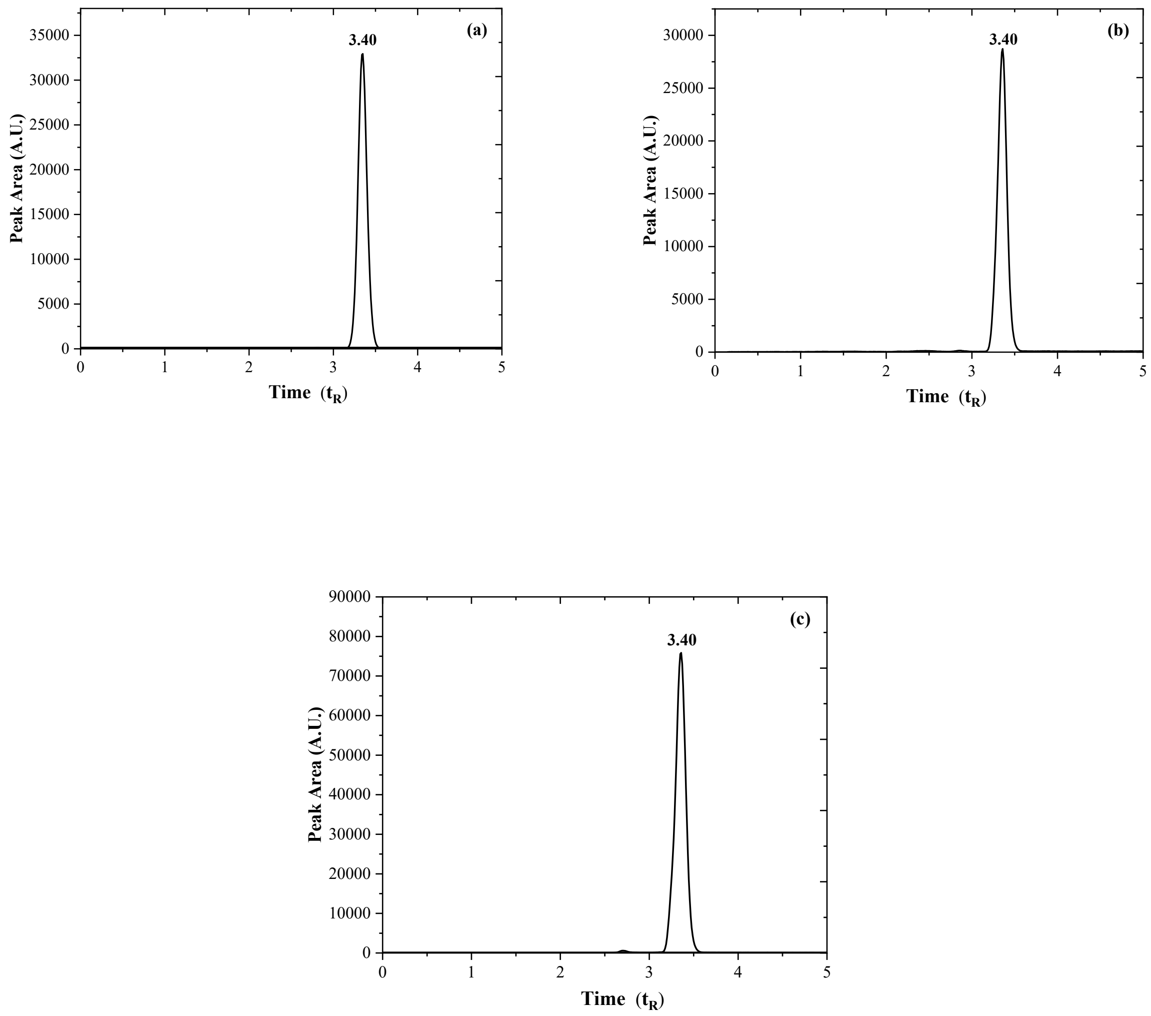


**Fig. S2.** Chromatograms of 5–FU in the presence of CDs ($1.25 \times 10^{-3}$ M): (a) α–CD, (b) β–CD, (c) γ–CD

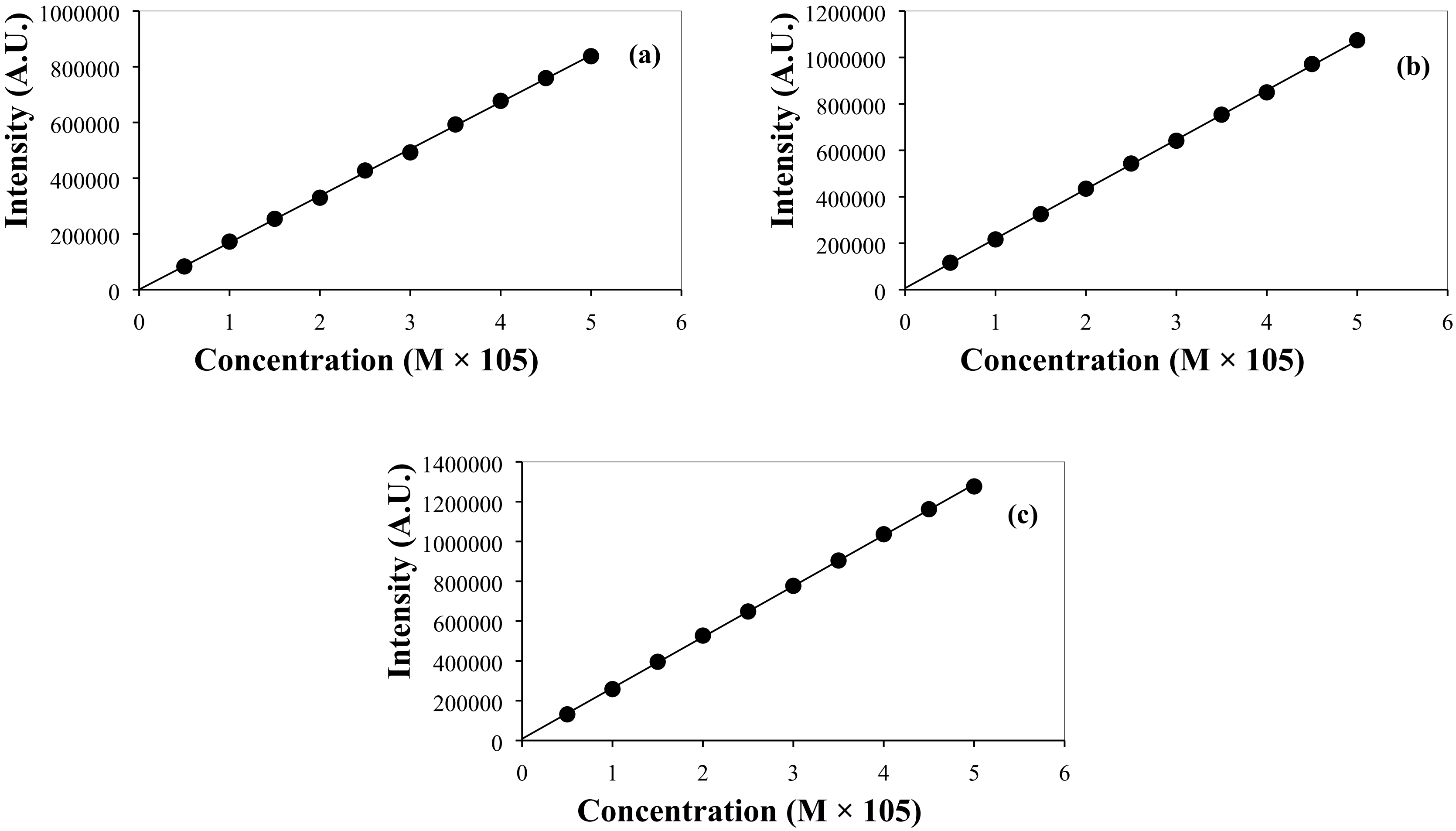


**Fig. S3.** Calibration curve of 5–FU in the concentration range of 0.50–5.00 × $10^5$ M in the presence of CDs (1.25 × $10^{-3}$ M): (a) α–CD, (b) β–CD, (c) γ–CD

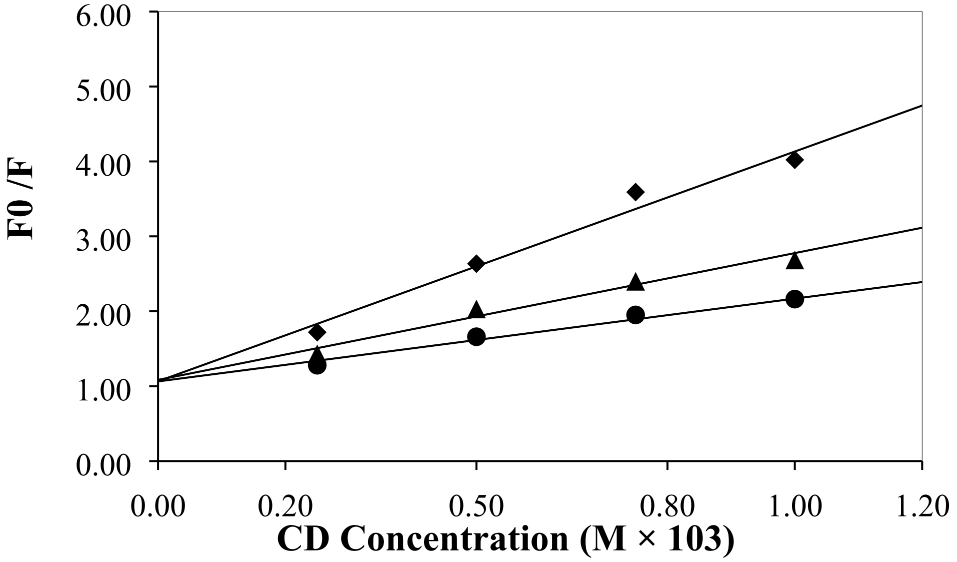


**Fig. S4.** Stern–Volmer plots for quenching of 5–FU by CDs (pH 7.0): α–CD (●), β–CD (▲) and γ–CD (♦)

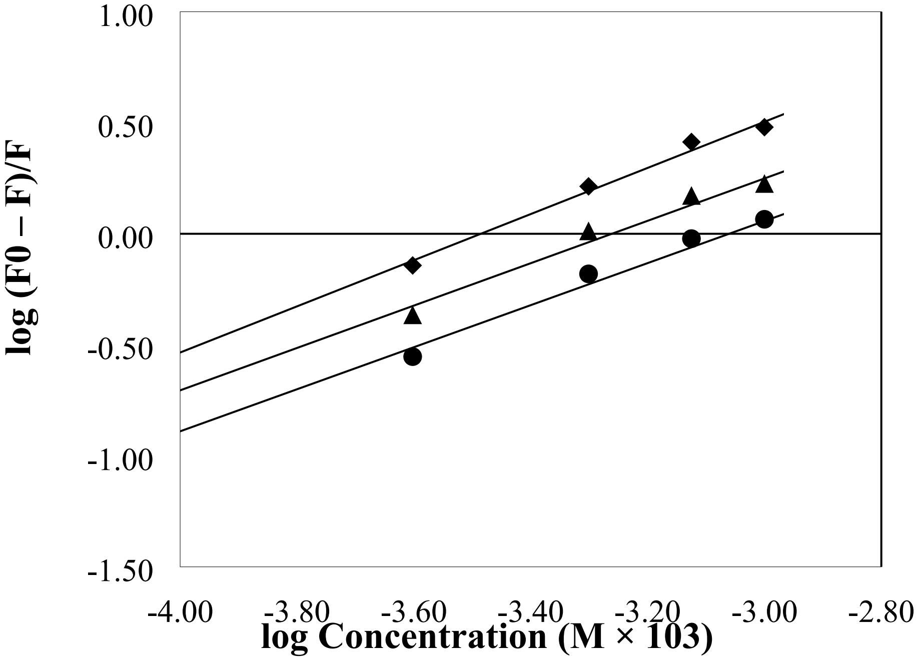


**Fig. S5.** A plot of log ($F_0$–F/F) versus log concentration of CDs (α–CD (●), β–CD (▲) and γ–CD (♦)) for quenching of the fluorescence of 5–FU by CDs at pH 7.0

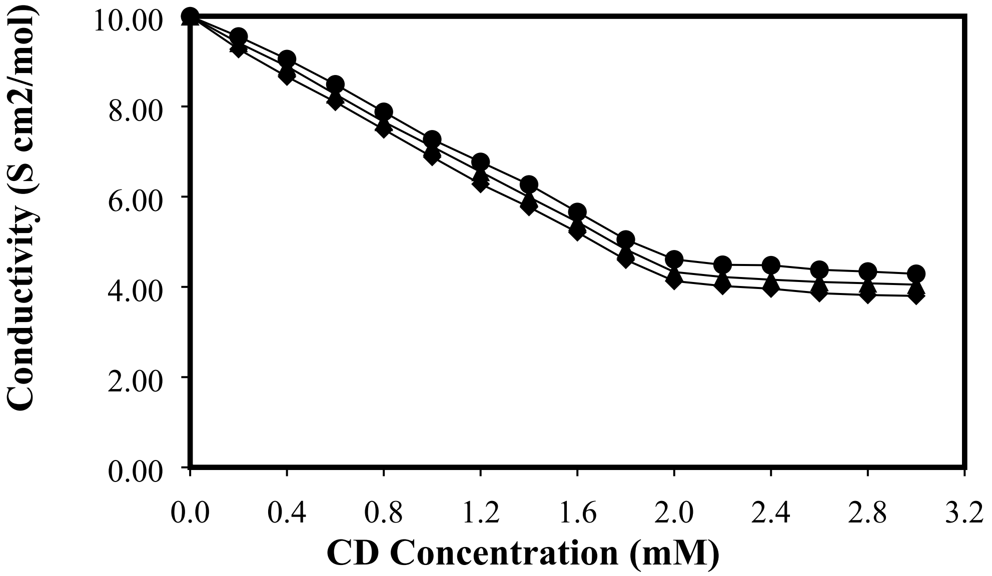


**Fig. S6.** Variations in molar conductivity of aqueous 5–FU ($2.0 \times 10^{-4}$M) solutions on increasing α– (●), β– (▲) and γ– (♦) CDs concentrations (0–3.2 mM) (25±1 $^{o}$C)

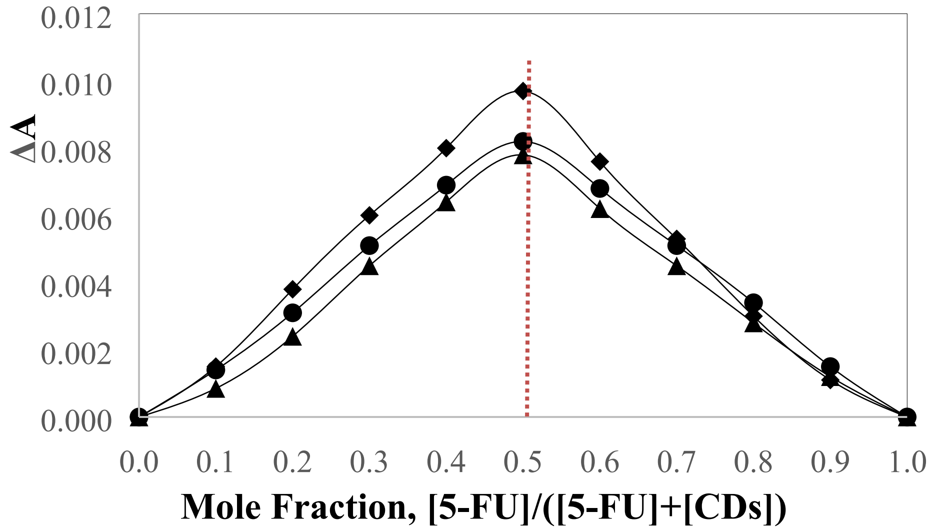


**Fig. S7.** Job's plot for 5-FU: CDs (1:1) complex in an aqueous solution at pH 7.0, where (●) 5–FU–α–CD, (▲) 5–FU–β–CD and (♦) 5–FU–γ–CD

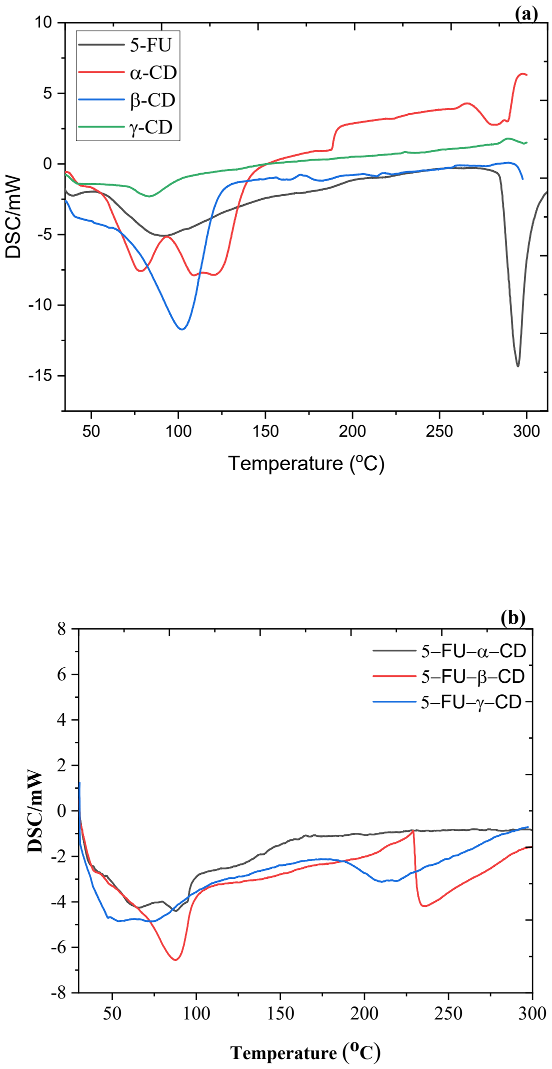


**Fig. S8.** DSC thermograms of (a): 5–FU, α–CD, β–CD, γ–CD, and (b): 5–FU–α–CD, 5–FU –β–CD, and 5–FU–γ–CD complexes

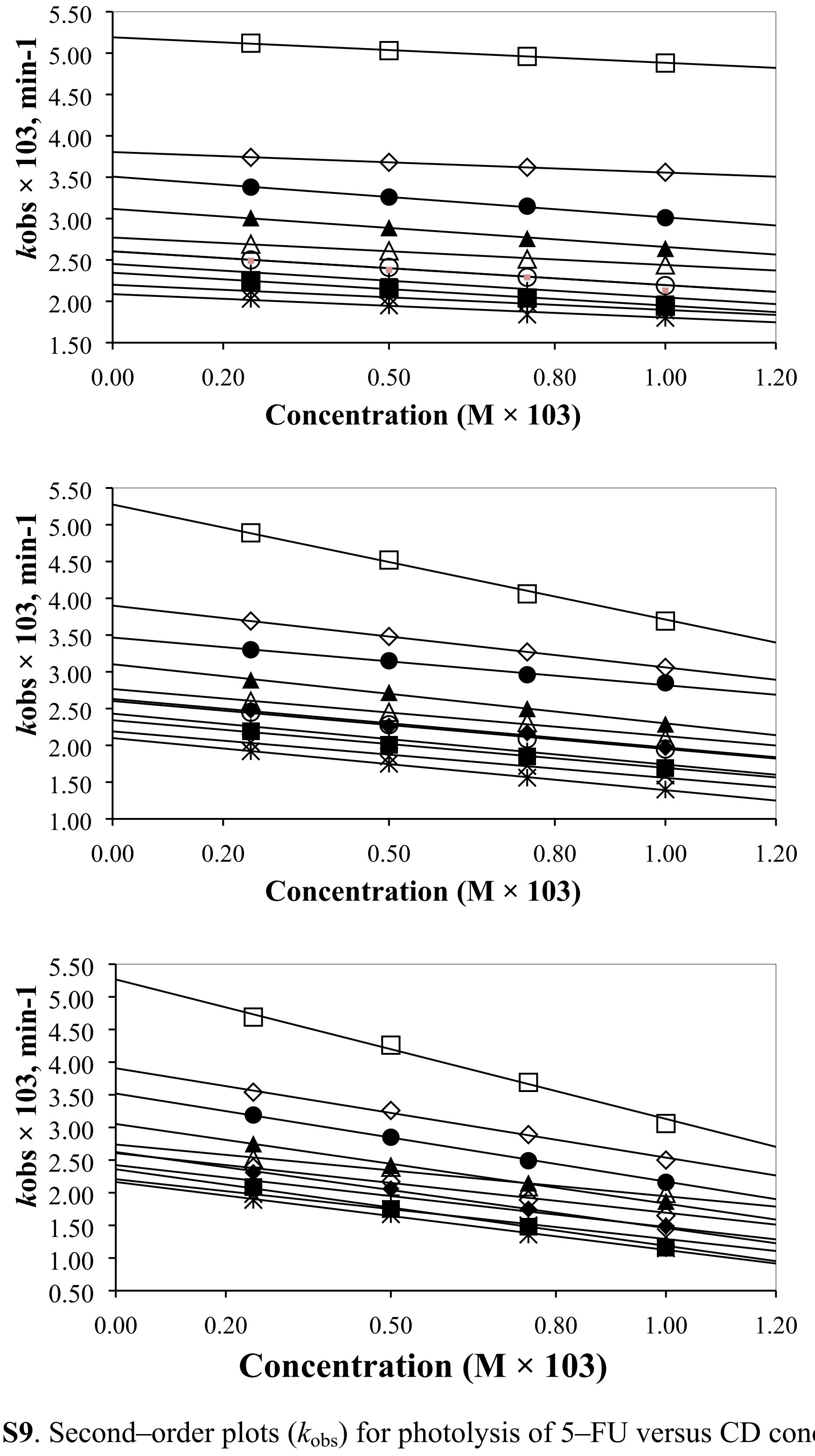


**Fig. S9**. Second–order plots ($k_{obs}$) for photolysis of 5–FU versus CD concentrations: α–CD (a), β–CD (b) and γ–CD (c) at pH: 2.0 (●); 3.0 (▲); 4.0 (♦), 5.0 (■); 6.0 (*); 7.0 (×); 8.0 (+); 9.0 (○); 10.0 (Δ); 11.0 (◊); 12.0 (□)

**Table. S1.** %Fluorescence loss of 5–FU in the presence of cyclodextrins at pH 7.0

| **CDs Concentration ($M \times 10^3$)** | **α–CD** | **β–CD** | **γ–CD** |
|---|---|---|---|
| 0.00 | 100.0 | 100.0 | 100.0 |
| 0.25 | 78.14 | 69.85 | 58.14 |
| 0.50 | 60.25 | 49.25 | 37.95 |
| 0.75 | 51.26 | 40.19 | 27.85 |
| 1.00 | 48.25 | 37.25 | 24.87 |
| 1.25 | 47.13 | 36.15 | 25.02 |

**Table S2.** % Entrapment efficiency of 5–FU in CDs

| **Concentration of CDs ($M \times 10^3$)** | **Entrapment Efficiency (%)** | | |
|---|---|---|---|
| | **α–CD** | **β–CD** | **γ–CD** |
| 0.00 | 0.00 | 0.00 | 0.00 |
| 0.25 | 21.86 | 30.15 | 41.86 |
| 0.50 | 39.75 | 50.75 | 62.05 |
| 0.75 | 48.74 | 59.81 | 72.15 |
| 1.00 | 53.75 | 62.75 | 75.13 |
| 1.25 | 57.85 | 63.85 | 76.98 |

**Table S3.** Details of peaks obtained from $^{1}H$ NMR

| Compounds | Chemical shift (δ, ppm) | Proton type | Splitting | Assignment |
|---|---|---|---|---|
| 5-FU | 7.6 | H6 | Doublet | Coupling of H6 with H7 |
| α–CD | 4.9-5.1 | H2-H3 | Doublet | α–linkage |
| | 3.6-3.9 | H4-H5 | Multiplets | Multiple hydrogens are present outside the cavity |
| | 3.4-3.7 | H6 | Triplet | Ring protons overlap |
| β–CD | 4.9-5.1 | H2-H3 | Doublet | α–linkage |
| | 3.7-3.9 | H4-H5 | Multiplets | Multiple hydrogens are present outside the cavity |
| | 3.4-3.7 | H6 | Triplet | Ring protons overlap |
| γ–CD | 4.9-5.1 | H2-H3 | Doublet | α–linkage |
| | 3.7-3.9 | H4-H5 | Multiplets | Multiple hydrogens are present outside the cavity |
| | 3.4-3.7 | H6 | Triplet | Ring protons overlap |
